# Different Stress-Induced Calcium Signatures Are Reported by Aequorin-Mediated Calcium Measurements in Living Cells of *Aspergillus fumigatus*

**DOI:** 10.1371/journal.pone.0138008

**Published:** 2015-09-24

**Authors:** Alberto Muñoz, Margherita Bertuzzi, Jan Bettgenhaeuser, Nino Iakobachvili, Elaine M. Bignell, Nick D. Read

**Affiliations:** 1 Manchester Fungal Infection Group, Institute of Inflammation and Repair, University of Manchester, Manchester, United Kingdom; 2 Fungal Cell Biology Group, Institute of Cell Biology, University of Edinburgh, Edinburgh, United Kingdom; 3 Centre for Molecular Bacteriology and Infection, Department of Medicine, Imperial College London, London, United Kingdom; Universidade de Sao Paulo, BRAZIL

## Abstract

*Aspergillus fumigatus* is an inhaled fungal pathogen of human lungs, the developmental growth of which is reliant upon Ca^2+^-mediated signalling. Ca^2+^ signalling has regulatory significance in all eukaryotic cells but how *A*. *fumigatus* uses intracellular Ca^2+^ signals to respond to stresses imposed by the mammalian lung is poorly understood. In this work, *A*. *fumigatus* strains derived from the clinical isolate CEA10, and a non-homologous recombination mutant *ΔakuB* ^KU80^, were engineered to express the bioluminescent Ca^2+^-reporter aequorin. An aequorin-mediated method for routine Ca^2+^ measurements during the early stages of colony initiation was successfully developed and dynamic changes in cytosolic free calcium ([Ca^2+^]_c_) in response to extracellular stimuli were measured. The response to extracellular challenges (hypo- and hyper-osmotic shock, mechanical perturbation, high extracellular Ca^2+^, oxidative stress or exposure to human serum) that the fungus might be exposed to during infection, were analysed in living conidial germlings. The ‘signatures’ of the transient [Ca^2+^]_c_ responses to extracellular stimuli were found to be dose- and age-dependent. Moreover, Ca^2+^-signatures associated with each physico-chemical treatment were found to be unique, suggesting the involvement of heterogeneous combinations of Ca^2+^-signalling components in each stress response. Concordant with the involvement of Ca^2+^-calmodulin complexes in these Ca^2+^-mediated responses, the calmodulin inhibitor trifluoperazine (TFP) induced changes in the Ca^2+^-signatures to all the challenges. The Ca^2+^-chelator BAPTA potently inhibited the initial responses to most stressors in accordance with a critical role for extracellular Ca^2+^ in initiating the stress responses.

## Introduction

*Aspergillus fumigatus* causes multiple human and animal diseases, the manifestations of which depend upon a complex interplay between pathogen- and host-mediated activities [1–3]. Due to their small size, airborne spores (conidia) can penetrate deep into lung alveoli; however, in healthy individuals inhaled spores are likely to be efficiently silenced by coordinated innate immune mechanisms including the involvement of macrophages and neutrophils [4]. Antifungal defences are impaired in immunocompromised individuals (e.g. recipients of allogenic hematopoietic stem cell- or solid organ-transplants) in whom fungal colonies can become established following spore inhalation [5–7]. This can give rise to the most lethal form of infection, invasive aspergillosis (IA), which annually causes around 200,000 deaths worldwide [8]. The total burden of aspergillus-related diseases, including chronic, semi-invasive and allergic disease, is estimated as being > 2 million cases annually in Europe alone [9]. In the case of pre-existing structural lung defects, fungal balls (or aspergilloma) can form in pulmonary cavities [10,11]. For patients with asthma, cystic fibrosis or immunological lung defects, disease manifests [12–14] as allergic broncopulmonary aspergillosis (ABPA), which might progress into chronic, semi-invasive, pulmonary aspergillosis (CPA). In comparison to colonised patients with similar underlying disease, CPA has been demonstrated to reach mortality rates of 42.8% over an observation period of 2 to 3 years [15].

Despite the potentially life-threatening consequences of *A*. *fumigatus*-related disease, the early stages of colony formation have not been studied in detail. When airborne spores enter the human body and initiate colony formation, they do so in challenging and dynamically changing microenvironments, containing multiple stressors [2]. Germinating spores may be exposed to host immune attack, heightened temperature, alkalinity, osmotic stress, fluctuations in the carbon dioxide to oxygen ratio, oxidative stress, mechanical perturbation, iron limitation or varying extracellular ion concentrations, including that of free Ca^2+^ [2,16].

Calcium is universally recognised as a crucial mediator of intracellular signalling in eukaryotes [17]. In filamentous fungi, it is well established that Ca^2+^-mediated signalling is important for hyphal growth and pathogenicity, but the mechanistic basis of its involvement is poorly understood. Several processes relevant to colony initiation in filamentous fungi were found to involve Ca^2+^-signalling. These include hyphal tip growth, hyphal branching and septation [18–21], tolerance of oxidative stress [22], mechanosensing [23], and spore germination [24]. In addition, Ca^2+^-mediated signalling and/or homeostasis is fundamental for *A*. *fumigatus* pathogenicity as shown, in a leukopenic model of infection, by the reduced virulence of isolates lacking: the voltage-gated Ca^2+^-channel Cch1 [25], the stretch-activated Ca^2+^-channel Mid1 [25], the vacuolar Ca^2+^-channel Yvc1 [25], the Ca^2+^-transporter PmcA [26], the catalytic subunit of calcineurin CalA/CnaA [18,19] or the Ca^2+^-responsive transcription factor CrzA [27,28]. Most of these mutants also show increased susceptibility to commercially available antifungal drugs. For instance deletion, mutation or inhibition of calcineurin activity enhances the sensitivity of *A*. *fumigatus* to echinocandins and nikkomicin Z [29,30]. Other compounds have been shown to display antifungal activity by targeting Ca^2+^-signalling, for example the antiarrhythmic drug amiodarone [31]. Calcium is therefore an integral component of the signal transduction network involved in colony initiation and the establishment of infection and thus may prove useful as a therapeutic target, either alone or in combination with existing compounds.

A routine method for cell population-level measurements of cytosolic free calcium ([Ca^2+^]_c_) dynamics was previously developed for living cells of filamentous fungi (*Aspergillus niger*, *Aspergillus awamori* and *Neurospora crassa*) using synthetic codon-optimized aequorin as a bioluminescent Ca^2+^-reporter [23]. The aims of the current study were to: (i) achieve useful levels of aequorin expression in *A*. *fumigatus* cells for routine [Ca^2+^]_c_ measurement by multiwell plate luminometry during the early stages of colony initiation; (ii) characterise the dynamic intracellular Ca^2+^-signatures produced by the fungus in response to a range of environmental stimuli that might be experienced by *A*. *fumigatus* during lung infection; (iii) determine the influence of extracellular Ca^2+^ and calmodulin on generating these Ca^2+^-signatures; and (iv) analyse the effects of these environmental challenges and pharmacological treatments on the growth of the fungus.

## Materials and Methods

### Strains and Culture Conditions

*Aspergillus fumigatus* strains used in this study are listed in Table 1. They were cultured at 25°C and 37°C in *Aspergillus* Minimal Medium (AMM) or *Aspergillus* Complete Medium (ACM) [32]. Conidia were harvested in sterile H_2_O from cultures grown at 37°C on solid ACM for 5 days and the conidial suspensions were filtered using Miracloth (Calbiochem). They were spun for 10 min at 4000 rpm, and washed twice with sterile H_2_O prior to enumeration using a Nikon Eclipse 80i microscope and a haemocytometer. Conidial counts were adjusted to 10^6^ conidia per ml for experimentation. Growth of pyrimidine auxotrophic transformants was supplemented with 50 mM uridine and 25 mM uracil.

**Table 1:** *A*. *fumigatus* strains used in this study.

Isolate	Genotype	Phenotype	Reference
CEA10 (CBS 144–89)	Clinical isolate	N/A	[33]
*ΔakuB* ^KU80^	*ΔakuB* ^ku80^::*pyrG* ^*Af*^ *-zeo*	5-FOA^S^	[34]
AEQ^CEA10^	*his2A* ^*t*^::[*gpdA* ^*P*^-*aeqS*-*ptrA*]	PtrA^R^	This study
AEQ^*ΔakuB*^	*akuB*::[*gpdA* ^*P*^-*aeqS*-*ptrA*]	Pyrimidine auxotroph, 5-FOA^R^	This study

5-FOA^S^ = Sensitive to 5-Fluoro-orotic acid. 5-FOA^R^ = Resistant to 5-Fluoro-orotic acid. *his2A* ^*t*^ = terminator region of the *A*. *fumigatus* histone 2A locus (intergenic to AFUA_3G05360 and AFUA_3G05370). [*gpdA* ^*P*^-*aeqS*-*ptrA*] = *A*. *fumigatus* aequorin expression cassette [*gpdA* promoter (*A*. *nidulans*)-synthetic aequorin-pyrithiamine resistance cassette (*A*. *oryzae*)]. PtrA^R^ = Resistant to pyrithiamine hydrobromide.

### Plasmid Construction

All plasmids used and generated are shown in Table 2. In all instances, the ampicillin resistance marker (*bla*) was used for selection and maintenance in bacterial cells. Oligonucleotides used to construct, and validate, the plasmids are indicated in Table 3. The synthetic codon-optimized apoaequorin gene (*aeqS*) was obtained from the plasmid pAEQ1-15 [23].

**Table 2:** Plasmids used and generated in this study.

Name	Purpose	Selection in *A*. *fumigatus*	Target locus	Reference
pAEQ1-15	Source of codon optimised *aeqS* gene	N/A	N/A	[23]
pSK379	Cloning vehicle for targeted insertion of aequorin cassette into *A*. *fumigatus* genome via pAEQ(I)	0.5 ug/ml pyrithiamine [*ptrA*]	Intergenic to AFUA_3G05360 and AFUA_3G05370 [*his2A* ^*t*^]	[35]
pAEQ(I)	Targeted insertion of *aeqS* gene into CEA10 genome	0.5 ug/ml pyrithiamine [*ptrA*]	Intergenic to AFUA_3G05360 and AFUA_3G05370 [*his2A* ^*t*^]	Derivative of pSK379, this study
pUC19	Cloning vehicle for targeted insertion of aequorin cassette into *A*. *fumigatus* genome via pAEQ(II)	N/A	N/A	
pAEQ(II)	Targeted insertion of *aeqS* gene into *ΔakuB* ^AKU80^ genome	1 ug/ml 5-FOA	AFUA_2G02620	Derivative of pUC19, this study

*amdS* = acetamidase gene of *A*. *nidulans*. Selection of transformants was performed onto minimal medium with only 0.01 M acetamide or acrylamide as a nitrogen source and lacking uridine.

**Table 3:** Oligonucleotides used in this study.

No.	Name	Sequence	Use
P1	GpdA_AEQ_F_GA	GAGCGACTATCTTTGCCCGGTGTATGAAACCG	Amplification of *gpdA* ^*P*^-*aeqS* from pAEQ(I)
P2	GpdA_AEQ_R_GA	TCTTGATCTTAGGGGACGGCACCGCCGTAGAG	Amplification of *gpdA* ^*P*^-*aeqS* from pAEQ(I)
P3	5FLANKSfiI_F_GA	CTCGGTAC***GGCCATATAGGCC***CATGAAGGCGC	Amplification of *ku80* ^5’^ from genomic DNA
P4	5FLANK_GpdA_R_GA	GCAAAGATAGTCGCTCCTCTAAATGGTTCAGA	Amplification of *ku80* ^5’^ from genomic DNA
P5	3FLANKSfiI_R_GA	TGCATGCC***GGCCATATAGGCC***CCCAGACACTG	Amplification of *ku80* ^3’^ from genomic DNA
P6	3FLANK_GpdA_F_GA	TCCCCTAAGATCAAGATGCTCTAGAATAGAAA	Amplification of *ku80* ^3’^ from genomic DNA
P7	AeqF	ATGACCTCCAAGCAGTACTCC	Amplification of *aeqS* from pAEQ1-15, Probe amplification for Southern blotting, Sequencing of pAEQ(I) and pAEQ(II)
P8	AeqR	TTAGGGGACGGCACCGCCGTA	Amplification of *aeqS* from pAEQ1-15, Probe amplification for Southern blotting, Sequencing of pAEQ(I) and pAEQ(II)
P9	gpdAF	GGTGATGTCTGCTCAAGCGG	Sequencing of pAEQ(I)
P10	gpdAR	TACTCCATCCTTCCCATCCC	Sequencing of pAEQ(I)
P11	pSK379AseqF1	TGGGGAGAGCAGGAAAATATG	Sequencing of pAEQ(I)
P12	pSK379AseqF2	CGGGATCCCATTGGTAACGA	Sequencing of pAEQ(I)
P13	pSK379AseqF3	ACACTCCTCGATTAGCCCTC	Sequencing of pAEQ(I)
P14	pSK379AseqR1	TGTGCAACGGCTAGACGGTT	Sequencing of pAEQ(I)
P15	pSK379AseqR2	AGGGTCATGCCTTCTCTCGT	Sequencing of pAEQ(I)
P16	M13F	GTTTTCCCAGTCACGAC	Sequencing of pAEQ(I)
P17	M13R	CAGGAAACAGCTATGAC	Sequencing of pAEQ(I)
P18	Ku80CheckF	TGTCGCCTAAAGGTTAGGGA	Sequencing of pAEQ(I)
P19	Ku80CheckR	CGACAAGACGGGATCAGATG	Sequencing of pAEQ(I)
P20	LucSBF	GTAACTACGCTCAACGTGTT	Sequencing of pAEQ(I)

Recognition sites for restriction enzymes are indicated by bold, underlined, italicised text.

To generate pAEQ(I), the *aeqS* gene was amplified by PCR from the plasmid pAEQ1-15 using the primers AeqF and AeqR. Subsequently, the *aeqS* cassette was blunt-ended, phosphorylated and ligated into PmeI-digested, dephosphorylated pSK379 [35,36]. The resulting pAEQ(I) plasmid contains a sequence encoding the promoter region (from -433 to -1 with respect to the ATG) of the constitutively expressed *Aspergillus nidulans* glyceraldehyde-3-phosphate dehydrogenase gene (ANIA_08041) designated *gpdA* ^*P*^; and a 1997 bp targeting sequence identical to the region intergenic to the *A*. *fumigatus* genes AFUA_3G05360 and AFUA_3G05370.

The plasmid pAEQ(II) was assembled using GeneArt technology and pUC19 (both from Invitrogen). The plasmid comprises the *aeqS* gene, also (as above) fused downstream of *gpdA* ^*P*^. This expression cassette [*gpdA* ^*P*^ *-aeqS*] was flanked by 800 bp of the 5’ and 3’ regions of the *akuB* ^KU80^ gene (AFUA_2G02620) to direct homologous recombination to the *akuB* ^KU80^ locus. The 5’ flank is identical to the genomic DNA sequence occurring at -800 to -1 bp of the region upstream of the *akuB* ^KU80^ ATG codon, and the 3’ flank is identical to the DNA sequence occurring at +188 to +988 bp of the region downstream of the *akuB* ^KU80^ stop codon [34]. The *gpdA* ^*P*^ *-aeqS* cassette was amplified by PCR from the plasmid pAEQ(I) using the oligonucleotides P1 and P2. Flanks were amplified by PCR using *A*. *fumigatus* genomic DNA and the oligonucleotides P3 and P4 or P5 and P6, for 5’ and 3’ respectively. Once purified via gel extraction, inserts were added to the linearized pUC19 vector and GeneArt reactions were performed according to the manufacturer’s instructions (Life Technologies). Oligonucleotides P3 and P6 included a SfiI restriction site in order to allow the excision of the gene replacement cassette.

Both plasmids were fully sequenced (S1 Data) and deposited into the collection of the Manchester Fungal Infection Group (pMFIG1 and pMFIG2).

### Generation of Aequorin-Expressing *A*. *fumigatus* Strains

*A*. *fumigatus* transformation was performed according to the protocol described in Szewczyk *et al*., 2007 [36]. For the AEQ^CEA10^ strain, *A*. *fumigatus* CEA10 protoplasts were transformed with circular pAEQ(I). Pyrithiamine resistant *A*. *fumigatus* transformants were selected by supplementing the media with 0.5 μg/ml pyrithiamine hydrobromide (Sigma) [37]. The presence and site of genomic integration of *aeqS* were verified by PCR using the oligos P7-P8 and P20-P8 respectively. Copy number was checked by Southern analysis. For this purpose, genomic DNA was digested with EcoRI and probed with an aequorin-specific hybridisation probe generated using the oligonucleotides P7 and P8. For the AEQ^*ΔakuB*^ strain, A. *fumigatus ΔakuB* ^KU80^ protoplasts were transformed with a gel-purified *ku80* ^*5’*^-*aeqS*-*ku80* ^*3’*^ cassette obtained by SfiI-mediated digestion of pAEQ(II). The original *ΔakuB* ^KU80^ used for this study was created by replacement of the *akuB* ^KU80^ locus (AFUA_2G02620) with the *pyrG* ^*Af*^ *-zeo* cassette [34]. As targeted integration of the *ku80* ^*5’*^-*aeqS*-*ku80* ^*3’*^ cassette would result in direct replacement of the resident *A*. *fumigatus pyrG* ^Af^ *-zeo* cassette, *A*. *fumigatus* transformants were selected by supplementing the media with 1 μg/ml 5-fluoroorotic acid (5-FOA), 5 mM uracil and 10 mM uridine (Sigma). The presence and site of genomic integration of *aeqS* were verified by PCR using the oligos P7-P8 and P18-P19 respectively. Copy number was checked by Southern analysis as described for AEQ^CEA10^. Both strains were deposited in the collection of the Manchester Fungal Infection Group (MFIG2 and MFIG3).

### Phenotypic Analyses

For phenotypic analysis on solid media, 10^3^ conidia (in 5 μl of distilled water) were inoculated onto AMM in triplicate. Radial growth of the aequorin-expressing strains was measured after 3 or 4 days at 25°C and compared to that of parental strains. Images were captured using a Nikon Coolpix 990 digital camera.

Conidial germ tube formation was imaged and quantified using a Nikon TE2000E inverted microscope with a 60x (1.2 NA) water immersion, plan apo objective (Nikon, Kingston-Upon-Thames, UK) and differential interference contrast (DIC) optics. For these analyses, 200 μl of a conidial suspension containing 10^6^ conidia per ml, in liquid AMM medium, was placed in each well of an eight-well slide culture chamber (Nalge Nunc International, Rochester, NY) and incubated at 37°C. Imaging and quantification of germinated conidia was performed at 5, 7, 9 and 11.5 h following inoculation. Three technical replicates were analysed for each time point and treatment. Percentages of conidial germination were calculated as an average ± SD for each strain.

To assess the impact of stress on the growth of mature germlings, fungal growth during the initial 96 h of incubation at 25°C was determined using a 96-well microtitre plate assay method by measurement of the optical density (OD) at 610 nm in a multimode plate reader (Berthold Technologies TriStar LB 941). 100 μl per well of a solution of 10^6^ conidia per ml in liquid AMM was incubated for 21 h in a clear 96-well plate with round bottomed wells. Three wells were analysed for each treatment. At the 21 h time point, 100 μl per well of medium inducing the different stressing conditions was added and then growth recorded at ~ 72 h post-stress. Results were calculated as an average ± SD for each experimental treatment.

### Protein Extraction and Western Blotting Analysis

For protein extraction, 10^6^ conidia were inoculated in 50 ml ACM and incubated at 25°C for 16 or 20 h. Mycelia were collected by filtering through Miracloth, snap-frozen in liquid nitrogen and lyophilised. The extraction of the proteins was performed as previously described [38]. The total protein concentration was determined using the commercially available bicinchoninic acid assay (BCA) assay (Sigma) according to manufacturer's instructions. Protein samples were separated on 15% SDS-PAGE gels. Recombinant aequorin was detected using a polyclonal rabbit anti-aequorin antibody (1:2000, Abcam) coupled with an HRP conjugated goat anti-rabbit IgG antibody (1:10000, Santa Cruz, USA).

### Analysis of [Ca^2+^]_c_ Dynamics following Exposure to Stressors

Conidia of the *A*. *fumigatus* strains expressing AeqS were suspended in liquid AMM containing 2.5 μM (AEQ^CEA10^) or 5 μM (AEQ^*ΔakuB*^) of the co-enzyme coelenterazine (Biosynth AG, Rietlistr, Switzerland). A 100 μl cell suspension was dispensed to each well of a white 96-well microtitre plate (Thermo Fischer, United Kingdom). Each treatment was performed as six replicates in the same multiwell plate. The plate was wrapped in aluminium foil to protect the light-sensitive co-enzyme and incubated at 25°C for 15 h, 18 h, 21 h or a maximum of 24 h for AEQ^CEA10^ or 28 h for AEQ^*ΔakuB*^ strains. A multimode plate reader (Berthold Technologies TriStar LB 941) was used to measure bioluminescence at 25°C over a time course of 12 min, taking measurements of the relative light units (RLUs) per well for 84 cycles (a cycle being the time it takes to measure all wells in the experiment). For each cycle the standard measurement time per well was 1 second and the standard cycle time was ~ 7.5 sec for all luminometry. During the eighth measurement cycle 100 μl of a stress-inducing solution was injected into relevant wells. Different stress treatments were applied by injecting 100 μl of the stimulating solutions to reach the following final concentrations of stressors: (1) isotonic AMM (mechanical perturbation), (2) AMM diluted in dH_2_O [52.5% v/v] (hypo-osmotic shock), (3) 5 mM, 20 mM or 200 mM of CaCl_2_ in AMM (high external Ca^2+^), (4) 2.5 mM, 5 mM, 10 mM and 20 mM H_2_O_2_ in AMM (oxidative stress), (5) 12.5%, 25% or 50% v/v human serum diluted in AMM (exposure to human serum), or (6) 50 mM, 250 mM or 500 mM mannitol in AMM (hyper-osmotic stress). The Ca^2+^-chelator 1,2-bis-(o-aminophenoxy)-ethane-N,N,N’,N’-tetraacetic acid) tetrapotassium salt (BAPTA) (5 mM) and the calmodulin antagonist trifluoroperazine (TFP) (50 μM) were obtained from Sigma (St. Louis, MO, USA). Stock solutions were prepared in water as appropriate and BAPTA and TFP were added as a pretreatment for 30 min prior to exposure to each stress condition.

### Calibration of Aequorin Bioluminescence

To convert relative light units (RLUs) into dynamic measurements of micromolar [Ca^2+^]_c_ in populations of conidial germlings during an experiment, the following empirically derived equation from Bonora *et al*., 2013 [39] was used:

$$Ca^{2+}\left(\mu M\right)=\frac{{\left(\frac{L}{L_{\max}}\times \lambda \right)}^{\frac{1}{n}}+\left({\left(\frac{L}{L_{\max}}\times \lambda \right)}^{\frac{1}{n}}\times K_{TR}\right)-1}{K_{R}-\left({\left(\frac{L}{L_{\max}}\times \lambda \right)}^{\frac{1}{n}}\times K_{R}\right)}\times 10^{6}$$ [Eq 1]

Where the mathematical constants *K*_*R*_ (= 7230000), *K*_*TR*_ (= 120), *λ* (= 1) and *n* (= 2.99) refer, respectively, to the Ca^2+^-bound and-unbound states of aequorin, aequorin consumption at saturating [Ca^2+^] and the number of Ca^2+^ binding sites on aequorin molecule, all of which are assumed to be identical to values previously described [39]. *L* is defined as background subtracted light intensity of an empty well at the sampling time point, and *L_max,_* defined in [Eq 2], is the cumulative total of light emitted, between time zero and the sampling time point. The *L*_*max*_ value at any particular cycle *N* (*L*_*max*_^*N*^) was calculated by subtracting the cumulative total of incrementally measured light emissions (*iL*), as measured up until the sampling time point (*N)*, from the sum total of detected light (*Total RLUs*) per experiment. Thus:

$$L\max^{N}=Total\;RLUs-\overset{N}{\underset{i=1}{\sum }}iL^{N}$$ [Eq 2]

The quantification of *Total RLUs* was performed subsequent to all other data collections, via complete discharge of the total cellular aequorin, achieved by injecting 100 μl of a discharge solution (3 M CaCl_2_ in 20% ethanol) into each well in six parallel wells at the end of the experiment. The discharge solution permeabilizes the fungal plasma membrane allowing entry of an excess of CaCl_2_. At this point a further capture of RLUs was conducted over a similar n = 84 measurement cycle. Total RLUs emitted by a sample at discharge was calculated as the sum of (n = 84) incrementally measured light emissions (*iL*) each of which was calculated as:

$$iL=\left(t_{N}-t_{N-1}\right)\times \left(\frac{L_{N}-L_{N-1}}{2}\right)$$ [Eq 3]

where *N* = measurement cycle, *t* = time, and *L* = background subtracted light intensity at sampling time. A correction factor of 1.24 was applied to account for ethanol-mediated quenching of aequorin luminescence [23]. Therefore,

$$Total\;RLUs=\overset{84}{\underset{N=1}{\sum }}iL\times 1.24$$ [Eq 4]

An Excel spreadsheet for the conversion of RLUs into [Ca^2+^]_c_ using the above formulae has been included as S2 Table, and an exemplar dataset (mechanical stress) has been included as S3 Table.

## Results

### Construction and Characterisation of *A*. *fumigatus* Aequorin-Expressing Isolates

To assess the utility of aequorin as a tool for measuring [Ca^2+^]_c_ dynamics in *A*. *fumigatus*, we constructed a Ca^2+^-reporter strain in two different *A*. *fumigatus* genetic backgrounds, the wild-type clinical isolate CBS 144–89 (CEA10) and a non-homologous end re-joining mutant *ΔakuB* ^KU80^, a null mutant in the AFUA_2G02620 locus, which exhibits significantly heightened homologous integration frequencies compared to non-mutated *A*. *fumigatus* isolates [34].

The Ca^2+^-reporter strain in the wild-type clinical isolate CEA10, named AEQ^CEA10^, was constructed via transformation with circular pAEQ(I) (S1 Fig). This vector contains a sequence of 1997 bp (termed *his2A* ^*t*^) which is identical to that occurring immediately downstream of the AFUA_3G05360 (*his2A*) stop codon, thereby directing targeted insertion of the plasmid into the *A*. *fumigatus* genome (S1 Fig).

Using the same strategy and the pAEQ(I) vector, initial attempts at producing an *A*. *fumigatus* aequorin-expressing strain in the non-homologous end re-joining mutant *ΔakuB* ^KU80^ failed repeatedly. Although viable transformants, having undergone homologous integration of the plasmid were obtained, AeqS expression was undetectable. Further analysis indicated that the pAEQ(I) construct was unsuitable for *ΔakuB* ^KU80^ strain construction due to spontaneous rearrangement of the target locus in 100% of transformants, leading to the excision of the *gpdA* ^*P*^ and a 5’ region of the AeqS coding sequence (data not shown). For the *ΔakuB* ^KU80^ background, we therefore took an alternative approach (S2 Fig) using the plasmid pAEQ(II), and exploiting the counter-selectable nature of pyrimidine auxotrophy. This involved the replacement of the *pyrG* ^*Af*^ *-zeo* cassette resident at the *akuB* ^KU80^ locus (AFUA_2G02620) with the aequorin cassette (S2 Fig). As the resultant aequorin expressing isolate, named AEQ^*ΔakuB*^, maintains a *ΔakuB* ^KU80^ genotype, the non-homologous end-joining phenotype remains exploitable and can therefore be used for subsequent mutant constructions. The expression of recombinant AeqS in the AEQ^CEA10^ and AEQ^*ΔakuB*^ isolates was verified by western blotting using an aequorin specific antibody (S3 Fig).

To ensure normal rates and efficiency of germination in the recombinant isolates, the germination rates of the parental isolates CEA10, *ΔakuB* ^KU80^, and the two aequorin expressing strains (AEQ^CEA10^ and AEQ^*ΔakuB*^) were assessed by incubation for 5, 7, 9 and 11.5 h in AMM at 37°C in eight-well slide culture chambers in the presence of the aequorin substrate coelenterazine at 2.5 or 5.0 μM (see Materials and Methods).

*Aspergillus fumigatus* conidia undergo isotropic growth before becoming polarized to produce a germ tube. The swollen conidia were classified as “germinated” as soon as they became polarised and a germ tube protrusion was visible. Relative to progenitor isolates, a slight delay in germination was observed in the aequorin transformants at the 7 and 9 h time points of growth at 37°C (S4 Fig and S4 Table). However, after 11.5 h the germination of all strains was more-or-less equivalent: 98 ± 0.3% in the CEA10 strain, 99 ± 0.8% in the AEQ^CEA10^, 99 ± 0.6% in the *ΔakuB* ^KU80^ strain, and 94 ± 1.2% in the AEQ^*ΔakuB*^ strain (S4 and S5A Figs).

The growth rate and morphology of the aequorin expressing strains and respective parental isolates were compared after growth on agar plates in AMM at 37°C (S5 Fig). Plates were inoculated with 10^3^ conidia in 5 μl of water and the colony diameters were measured at 48 h post-inoculation (S5 Fig). When supplemented with 50 mM uridine and 25 mM uracil, the auxotrophic AEQ^*ΔakuB*^ strain achieved growth comparable to wild type levels. Therefore, for all experiments involving AEQ^*ΔakuB*^, and for all growth temperatures reported in this study, supplementation of the medium with 50 mM uridine and 25 mM uracil was used.

### Quantitation and Optimisation of [Ca^2+^]_c_ Concentration Measurements

**Fig 1:**
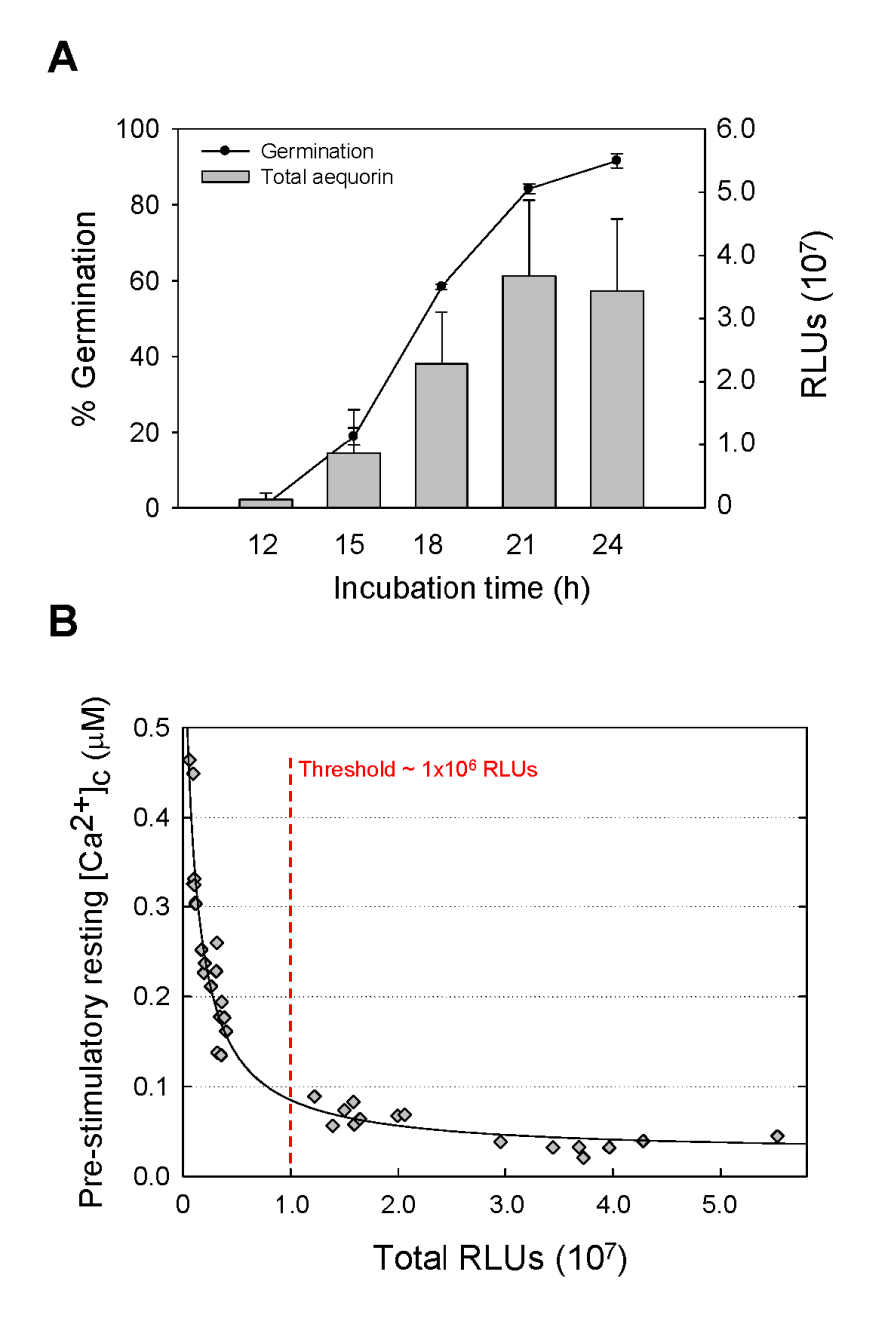
Increased intracellular aequorin correlates with the extent of conidial germination and fungal biomass. (A) Percentages of germination and total cytosolic aequorin present (in arbitrary relative light units, RLUs), as measured by using the aequorin discharge protocol (see Materials and Methods), for the AEQ^CEA10^ strain. (B) Influence of the total amount of aequorin produced by fungal cells on the calculated pre-stimulatory resting [Ca^2+^]_c_ level.

AEQ^CEA10^ spore germination and germ tube growth results in a proportional increase in fungal biomass which is reflected by an increase in the total aequorin present in the cells (Fig 1A). Time-course analysis of AEQ^CEA10^ spore germination for a period of 24 h at 25°C in AMM showed that > 80% of the conidia had germinated after 21 h of incubation (Fig 1A). This correlated with the maximal amount of aequorin expression in germlings, as measured by the maximum amount of aequorin luminescence detected (Fig 1A).

The [Ca^2+^]_c_ concentration prior to stimulation, known as resting [Ca^2+^]_c_, in eukaryotic cells, is typically between 0.05 and 0.1 μM [40]. In order to determine the minimum level of aequorin expression required to produce reliable measurements of [Ca^2+^]_c_ in the 0.05–0.1 μM concentration range, we discharged all of the aequorin present in ten independent aequorin expressing transformants (obtained in both of the CEA10 and the *ΔakuB* ^KU80^ backgrounds) at a range of developmental time-points, temperatures and in different media (S1 Table). These total RLUs values were then plotted against the pre-stimulatory resting level of [Ca^2+^]_c_ concentration (as calculated by using Eq 1). Fig 1B shows that the calculations of the pre-stimulatory [Ca^2+^]_c_ resting level conforms to the expected range of 0.1–0.05 μM when the Total RLUs were > ~ 1 x 10^7^ RLUs, but becomes unreliable at RLU values lower than this threshold. Thus, we concluded that the amount of aequorin expressed in conidia and conidial germ tubes at ≤ 15 h of incubation is too low (as shown by the Total RLUs at the 15 h time point in Fig 1A) to obtain reliable [Ca^2+^]_c_ measurements under the culture conditions used in this study

### Ca^2+^-Signatures in Response to Hypo-Osmotic Shock and Mechanical Perturbation Are Growth-Dependent

**Fig 2:**
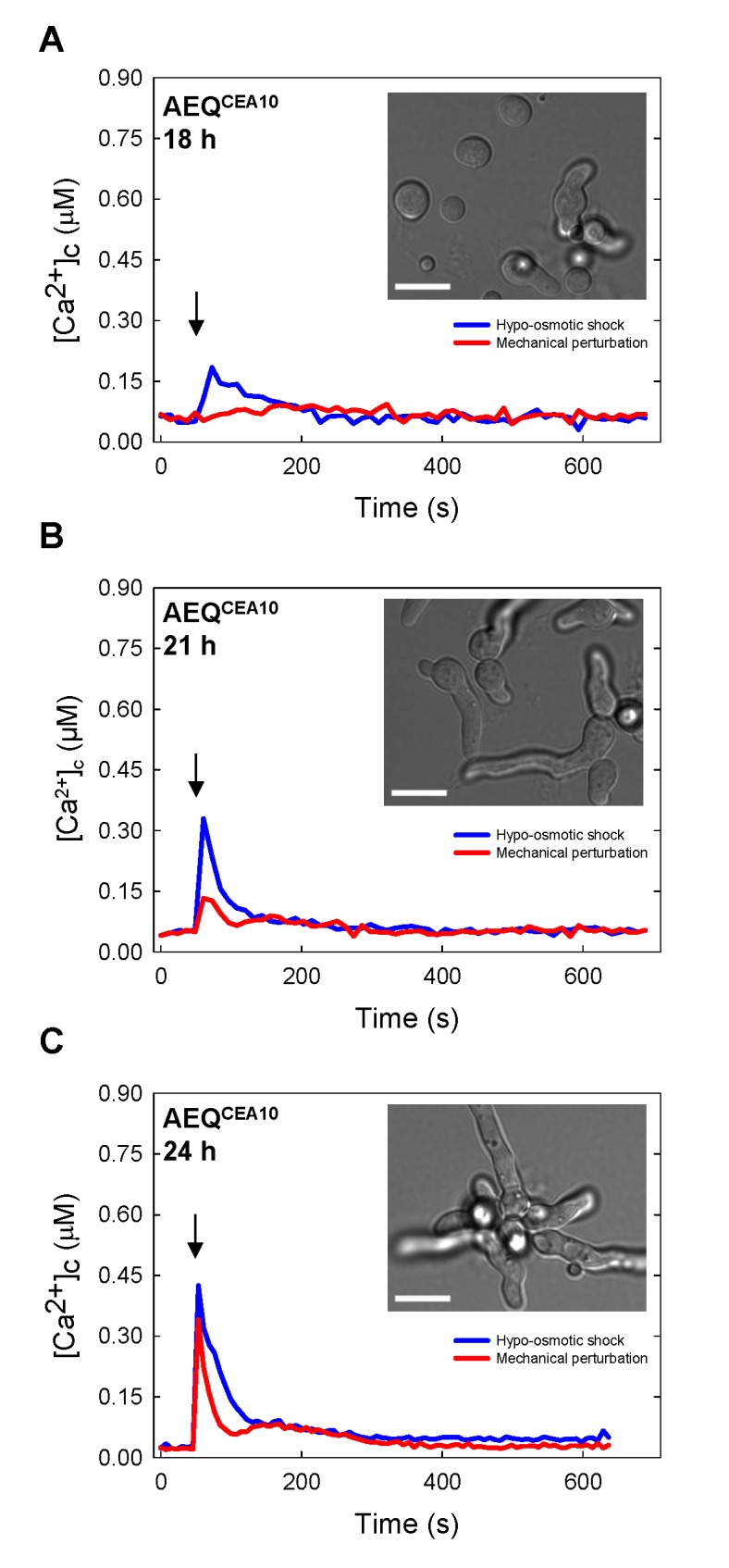
Ca^2+^-signatures in response to mechanical perturbation and hypo-osmotic shock are growth dependent. (A-C) The aequorin expressing strain AEQ^CEA10^ was subjected to each stressor at various time-points of growth (18 to 24 h) at 25°C. Cultures were also microscopically analysed in order to compare the stage of conidial germination and germ tube growth with the [Ca^2+^]_c_ response. For clarity, average values for six technical replicates are shown without error bars; however the data is plotted with SD error bars in S6 Fig for comparison. The arrows indicate the point at which each stress was applied via the injectors of the plate reader. Bar: 10 μm.

Previous studies involving aequorin-based, multiwell plate luminometry measurements in *A*. *awamori* and *N*. *crassa* have demonstrated that transient [Ca^2+^]_c_ increases, with reproducible Ca^2+^-signatures, are induced when these fungi are: (i) mechanically perturbed (by a rapid injection of iso-osmotic growth medium); (ii) exposed to hypo-osmotic shock (by injecting diluted 5% growth medium); or (iii) treated with a high (0.05–50 mM) concentration of external Ca^2+^ (by injecting growth medium containing these different concentrations of Ca^2+^ [23,31,41–43]. In this study, a plate luminometer incorporated into a multimode plate reader, and having built-in injectors, was utilised to subject *A*. *fumigatus* aequorin-expressing isolates to similar physiochemical perturbations. Each of these environmental challenges was found to produce a unique, and reproducible, Ca^2+^-signature in *A*. *fumigatus* (Fig 2 and S6 Fig).

In response to mechanical perturbation and hypo-osmotic shock the dynamic nature and magnitude of the Ca^2+^-signature was found to correlate directly with the growth stage of the conidial germlings (Fig 2 and S6 Fig). The amplitude of the [Ca^2+^]_c_ response was greater for hypo-osmotic shock than mechanical stress and increased as conidial germlings matured. A distinct [Ca^2+^]_c_ transient in response to hypo-osmotic shock was detectable after 18 h of incubation and successively increased in amplitude after 21 and 24 h, respectively (Fig 2). After 24 h of incubation the amplitude of the [Ca^2+^]_c_ response to hypo-osmotic shock was 0.42 μM. In contrast, [Ca^2+^]_c_ responses to mechanical perturbation were only observed after 21 h and the amplitude of the [Ca^2+^]_c_ response increased to a maximum of 0.35 μM after 24 h of incubation. The amount of spore germination increased from ~ 60% at 18 h to ~ 90% at 24 h of incubation (Fig 1A). Thus the increased amplitudes of the Ca^2+^-signatures in response to hypo-osmotic shock and mechanical perturbation are growth-dependent. The AEQ^*ΔakuB*^ strain also showed a growth-dependent [Ca^2+^]_c_ response to hypo-osmotic shock and mechanical perturbation, the magnitude of which was similar to that observed in AEQ^CEA10^ in terms of the extent of germination and germ tube growth (S7 Fig). However longer incubation times were needed to achieve a maximal [Ca^2+^]_c_ response which was coincident with the increase in incubation time that was necessary to achieve equivalent levels of germination and germ tube lengths (see insets in. S7 Fig). Having optimised the method, subsequent analyses of stress responses were primarily conducted with the AEQ^CEA10^ isolate.

### Responses to Extracellular Stresses Are Dose- and Stimulus-Dependent

The main focus of this study was on sensory perception and responses to stressors during the initiation of fungal infection. During mammalian infection the infecting fungal cell must balance, and appropriately integrate, homeostatic and stress-responsive adaptations. In order to assess the environmental challenges that fungal cells might encounter during lung infection, the following stimuli were studied: (1) hyper-osmotic shock (with 500 mM mannitol), (2) high extracellular Ca^2+^ (200 mM CaCl_2_), (3) oxidative stress (with 20 mM hydrogen peroxide); and (4) exposure to human serum (50%). In each case these environmental challenges were delivered by injecting modified growth medium into wells of the 96-well plate containing conidial germlings that had been pre-incubated, in the absence of stressor, for 21 h.

**Fig 3:**
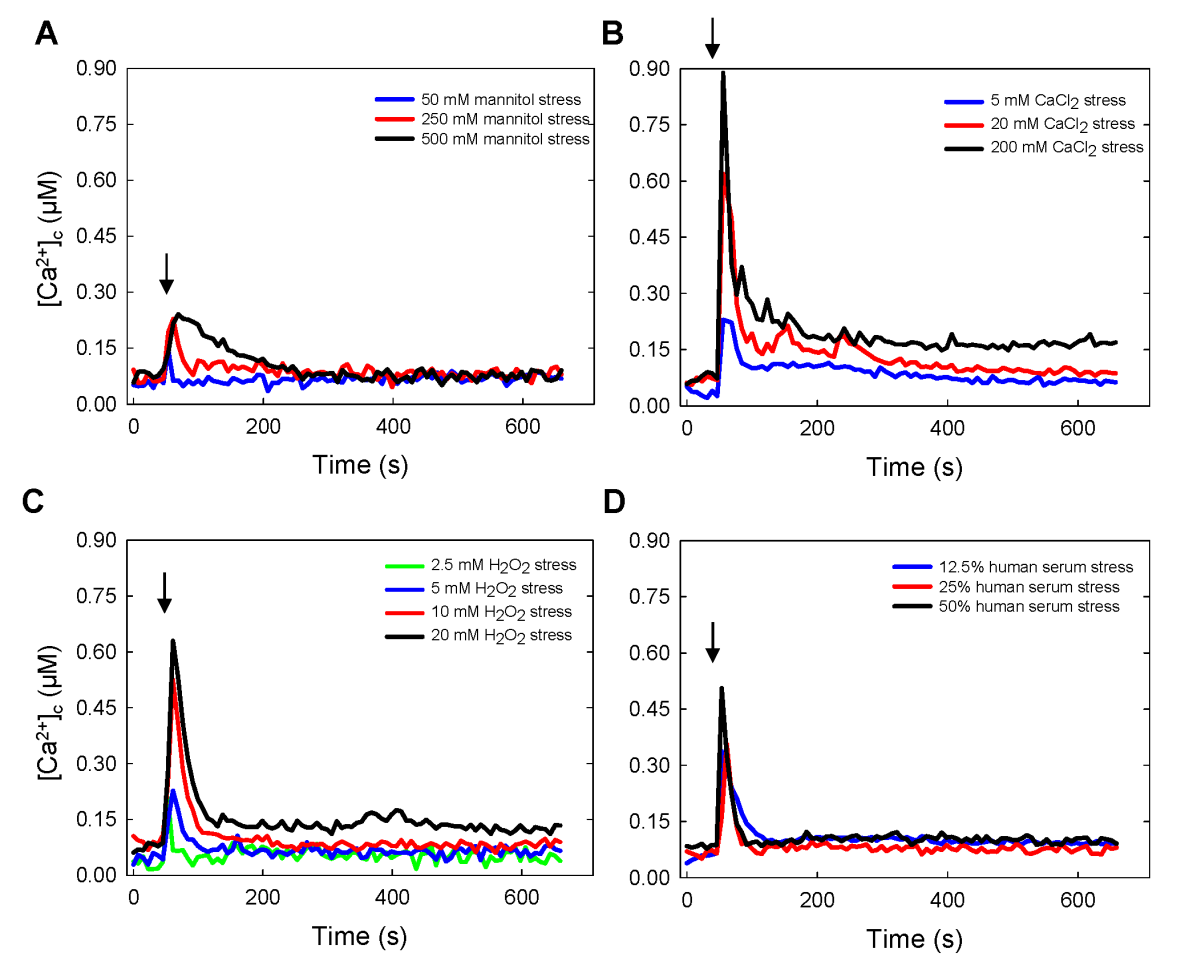
Dose and stress-dependent Ca^2+^-signatures in response to (A) mannitol (hyper-osmotic shock), (B) high extracellular Ca^2+^, (C) exposure to H_2_O_2_ (oxidative stress), and (D) exposure to human serum. After growth for 21 h at 25°C, *A*. *fumigatus* AEQ^CEA10^ strain was challenged with different stressors applied at points indicated by arrows at the final concentrations shown in the Figure. For clarity, average values are shown; however technical replicates are plotted in S8 Fig.

Each stressor prompted immediate and distinctly different [Ca^2+^]_c_ transients and thus different Ca^2+^-signatures (Fig 3 and S8 Fig), the amplitudes of which were dose-dependent with respect to each stressing stimulus. Following an initial rapid response to hyper-osmotic shock and serum treatments, there was a recovery of [Ca^2+^]_c_ to the pre-stimulatory [Ca^2+^]_c_ resting levels detected before the application of the stress stimulus. However, in response to high Ca^2+^ (5–200 mM) or oxidative stress (5–20 mM) the [Ca^2+^]_c_ did not fully recover to the pre-stimulatory resting levels within 10 min of the initial application of the stress stimulus (see below). The highest [Ca^2+^]_c_ amplitude was prompted by treatment with 200 mM extracellular CaCl_2_ (Fig 3B and S8 Fig), which provoked an increase in [Ca^2+^]_c_ to ~ 0.9 μM. The magnitude of responses from high to low was highest for the high extracellular Ca^2+^ treatment and smallest for mannitol challenge (Ca^2+^ > H_2_O_2_ > serum > mannitol). Similar results were also achieved using the AEQ^*ΔakuB*^ strain (data not shown).

Following a [Ca^2+^]_c_ increase, fungal cells activate Ca^2+^-pumps and-transporters in order to attempt to restore [Ca^2+^]_c_ to a resting level of 0.05–0.1 μM [40]. The kinetics of the [Ca^2+^]_c_ decreases following the application of each stressor varied between the different types of stimuli applied. After exposure to 500 mM mannitol (hypertonic shock), [Ca^2+^]_c_ slowly decreased to resting level over ~ 3 min (Fig 3A and S8 Fig). In contrast, [Ca^2+^]_c_ more rapidly decreased after stimulation with 200 mM CaCl_2_ or 20 mM H_2_O_2_, to a constant elevated level 0.15–0.2 mM but failed to regain the pre-stimulatory resting [Ca^2+^]_c_ (0.05–0.1 mM) within the 10 min of measurement (Fig 3B and 3C and S8 Fig).

### Calcium Modulatory Drugs Affect the Response to External Stimuli

A large number of Ca^2+^-signalling proteins and effectors, supporting the complexity of Ca^2+^ signalling and homeostasis in filamentous fungi, have been previously identified [44,45]. Two of the major components that commonly orchestrate Ca^2+^ signalling are extracellular Ca^2+^ and the Ca^2+^-binding protein calmodulin, which is a multipurpose, intracellular Ca^2+^ receptor that can relay [Ca^2+^]_c_ signals to regulate numerous cellular processes. In order to understand the respective roles played by extracellular Ca^2+^ and calmodulin during adaptation of *A*. *fumigatus* to extracellular stresses, two drugs were used to perturb stress responses: the cell impermeant Ca^2+^ chelator 1,2-bis-(o-aminophenoxy)-ethane-N,N,N’,N’-tetraacetic acid tetrapotassium salt (BAPTA) and the calmodulin antagonist trifluoperazine (TFP). BAPTA chelates Ca^2+^ ions in the extracellular medium thereby preventing the fungus from using extracellular Ca^2+^ during Ca^2+^-signalling. TFP, upon binding to calmodulin, induces a major conformational change in the Ca^2+^-calmodulin complex which results in its inability to interact with target enzymes [46].

**Fig 4:**
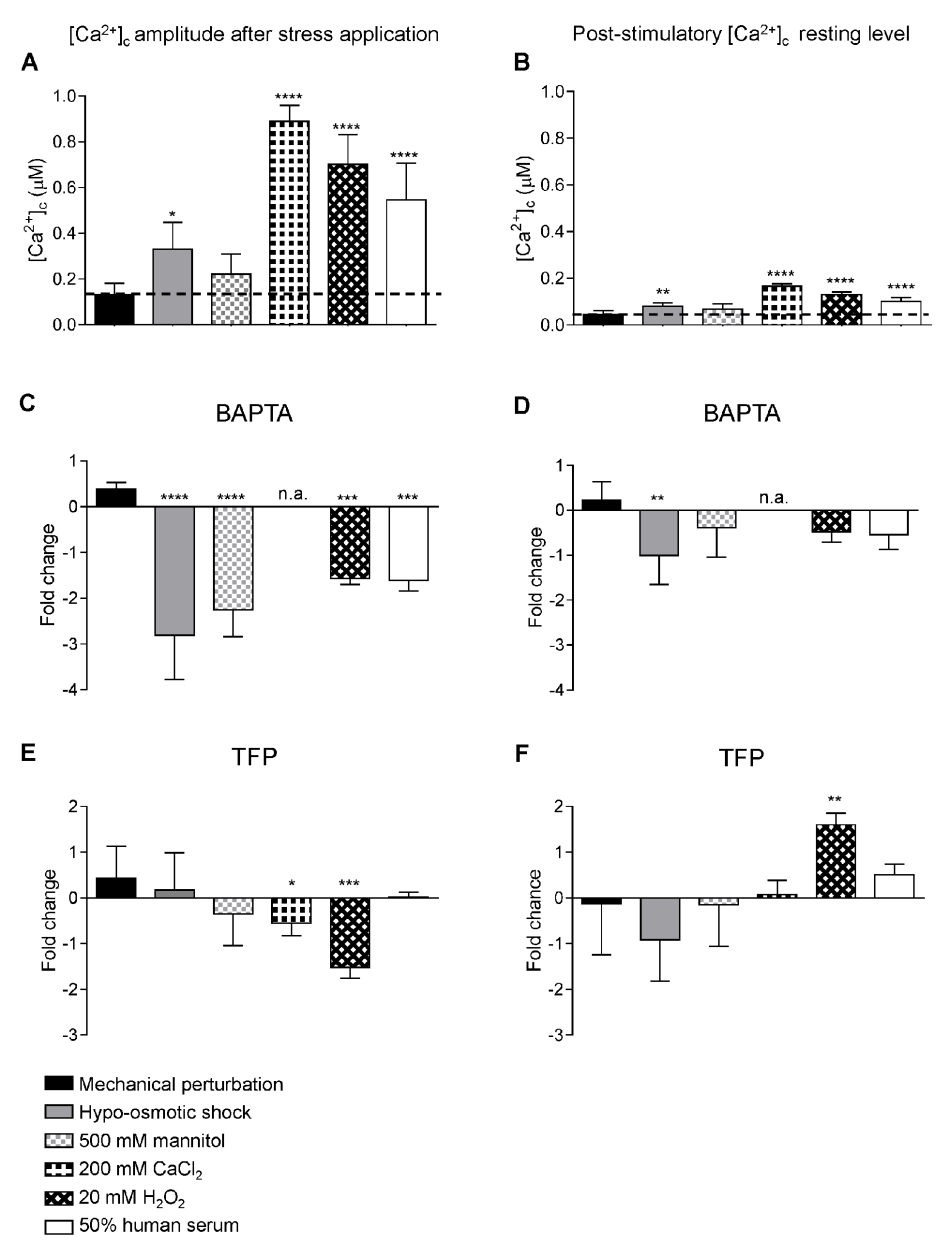
Pretreatment with the Ca^2+^-chelator BAPTA or the calmodulin inhibitor TFP differentially impacts upon Ca^2+^-signalling and homeostasis during *A*. *fumigatus* responses to stressors. After growth for 20.5 h at 25°C, *A*. *fumigatus* AEQ^CEA10^ cultures were pre-treated for 30 min with either 5 mM BAPTA or 50 μM TFP prior to challenge with stressors. [Ca^2+^]_c_ amplitudes (measured simultaneously to stress application) and post-stimulatory [Ca^2+^]_c_ resting levels (10 min after stress) are represented as [Ca^2+^]_c_ values (A-B) and as fold change (Log_2_ ratios of treated/untreated) with the modulators BAPTA (C-D) or TFP (E-F) under the different stress conditions indicated. For clarity, average values ± SD of the [Ca^2+^]_c_ amplitudes and post-stimulatory [Ca^2+^]_c_ resting levels are shown; however full Ca^2+^-signatures using the pretreatment with these modulators are plotted in S9 Fig Statistical analysis was performed using a 1-way ANOVA. * *p* < 0.05, ** *p* < 0.01, *** *p* < 0.005, **** *p* < 0.001.

The maximum [Ca^2+^]_c_ amplitude following application of the different stressors, and the post-stimulatory [Ca^2+^]_c_ resting levels at 10 min after stressor application, were analysed in untreated, BAPTA (5 mM) and TFP (50 μM) pre-treated samples (Fig 4 and S9 Fig). *A*. *fumigatus* showed very different [Ca^2+^]_c_ responses to each stressor with regard to [Ca^2+^]_c_ amplitude and post-stimulatory [Ca^2+^]_c_ resting levels (Fig 4 and S9 Fig). Pre-treatment with BAPTA or TFP radically altered [Ca^2+^]_c_ responses in both stressor- and drug-dependent ways. To standardise comparisons between treatments, variations in maximal [Ca^2+^]_c_ values were expressed as fold changes relative to challenge in the absence of drug (Fig 4C–4F). BAPTA pre-treatment significantly reduced the maximal [Ca^2+^]_c_ amplitude after exposure to hypo-osmotic shock (5% growth medium), hyper-osmotic shock (500 mM mannitol), oxidative stress (20 mM H_2_O_2_) and 50% human serum (Fig 4C and S9 Fig). Thus the [Ca^2+^]_c_ responses to these environmental challenges are likely to involve influx of extracellular Ca^2+^. The post-stimulatory [Ca^2+^]_c_ resting level rarely changed significantly, except in the case of hypo-osmotic shock where a > 1 fold reduction, relative to pre-stimulatory resting level, was observed (Fig 4D). The effect of BAPTA on exposure to high extracellular Ca^2+^ was not performed because the use of a Ca^2+^-chelator is incompatible with this type of treatment.

TFP pre-treatment prompted a significantly reduced [Ca^2+^]_c_ amplitude (relative to untreated cells) after exposure to high (200 mM) extracellular Ca^2+^ and oxidative stress (20 mM H_2_O_2_) suggesting that [Ca^2+^]_c_ responses are mediated by calmodulin in response to these treatments (Fig 4E and S9 Fig). Additionally, TFP pre-treatment led to a marked increase in the post-stimulatory [Ca^2+^]_c_ resting level following exposure to oxidative stress suggesting a role for calmodulin in regulating the return of the [Ca^2+^]_c_ concentration to its normal resting level (Fig 4F and S9 Fig) following oxidative stress.

**Fig 5:**
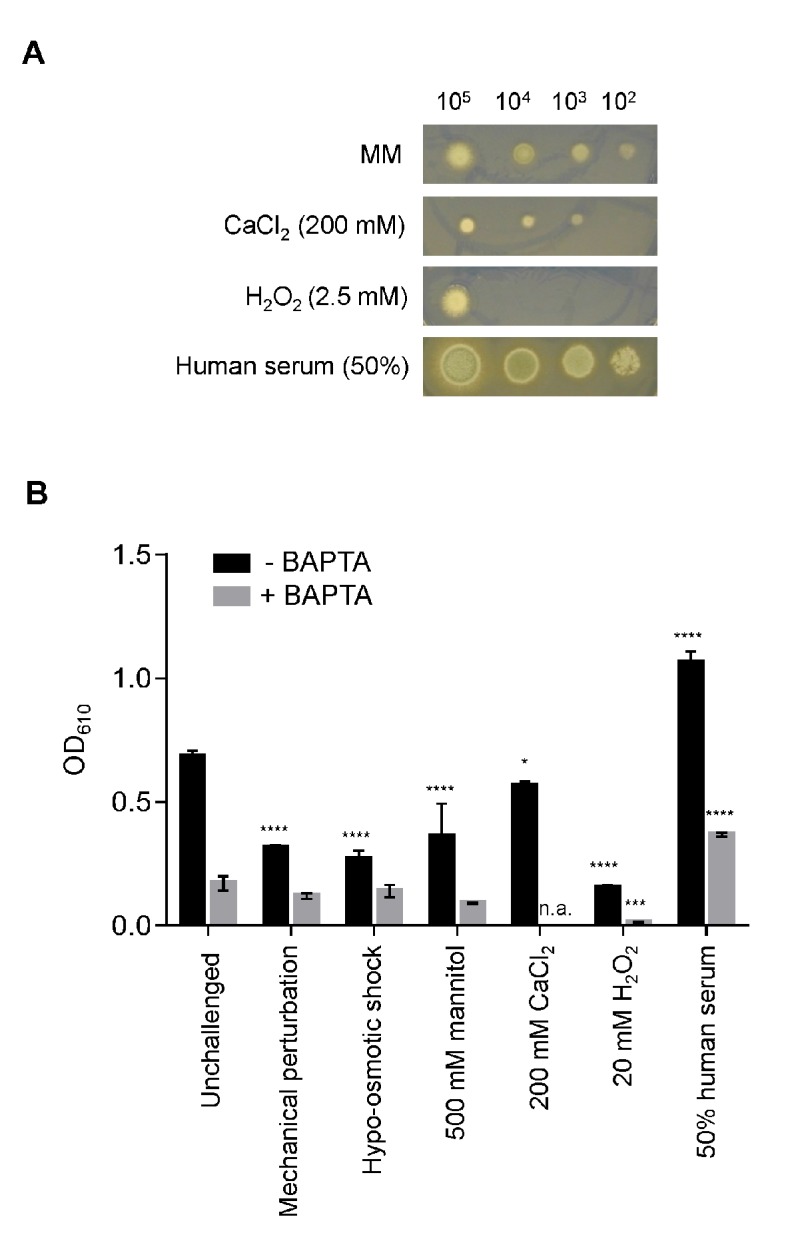
Growth of *A*. *fumigatus* is significantly impacted by the stressors and tolerance to oxidative stress is Ca^2+^ dependent. (A) Colonial growth phenotypes of a serial dilution (10^5^, 10^4^, 10^3^ and 10^2^ spores) of *A*. *fumigatus* AEQ^CEA10^ in AMM supplemented with 200 mM CaCl_2,_ 2.5 mM H_2_O_2_ and 50% human serum, 72 h at 25°C. (B) Optical density (OD_610_) of *A*. *fumigatus* AEQ^CEA10^, measured in the presence or absence of BAPTA, following challenge with stressors. Prior to the application of the challenge indicated, cultures were grown at 25°C for 21 h, or 20.5 h if pre-treatment with BAPTA was applied. Following challenge, growth was allowed to commence for a further 72 h before measurements were taken_._ Statistical significance was calculated using 2-way ANOVA to compare each challenge, in the presence or absence of BAPTA, to the respective unchallenged measurements. * *p* < 0.05, ** *p* < 0.01, *** *p* < 0.005, **** *p* < 0.001.

In order to assess the effects of stressors upon *A*. *fumigatus* growth, and the role of extracellular Ca^2+^ in adapting to such challenges, a phenotypic growth assay was implemented (Fig 5). To assess colonial phenotypes the different stressors were added to solid AMM medium, serial dilutions of spores (10^5^, 10^4^, 10^3^ and 10^2^ spores) in 5 μl of water were inoculated and colonial size and morphology evaluated after 72 h of growth at 25°C (Fig 5A). In a separate analysis aimed at understanding stresses applied in the same multiwell plate liquid AMM environment utilised for aequorin-mediated measurement of Ca^2+^-signalling (Fig 5B), the different stressors were applied to untreated and BAPTA pre-treated 21 h-old fungal cultures, and their growth monitored over the next 72 h. All stressors prompted significant growth perturbations when applied on solid (Fig 5A) or in liquid (Fig 5B) media. Growth was greatly enhanced by addition of 50% human serum, consistent with one or more components of this complex substrate providing nutrients for the growing pathogen (Fig 5B). This observation was also supported by analyses of radial growth which indicated more robust growth in response to serum (Fig 5A). In contrast, fungal growth was highly sensitive to 2.5 mM H_2_O_2_ and concentrations higher than 2.5 mM were found to be toxic to the cells in the 21–72 h period between stimulation and growth measurement (Fig 5). Highlighting the importance of extracellular Ca^2+^ for *A*. *fumigatus* growth (Loss and Bertuzzi *et al*., submitted), BAPTA treatment decreased hyphal growth rate in liquid culture in the absence of a challenge. Most importantly, consistent with a role for extracellular Ca^2+^ in mediating the response of *A*. *fumigatus* to these stresses, BAPTA treatment significantly perturbed hyphal growth rate in liquid culture when hyphae were challenged with H_2_O_2_ and 50% human serum (Fig 5B). In the presence of BAPTA, oxidative stress and human serum caused approximately a 10 fold decrease and a 2 fold increase respectively, relative to unchallenged *A*. *fumigatus* fungal growth (Fig 5B). Similar experiments were also performed in the presence of the calmodulin inhibitor TFP. However, no significant inhibition/modulation of the fungal growth by TFP was observed during the 21–72 h period (data not shown). This observation of the differential growth inhibitory effect of BAPTA and TFP is consistent with our observations that showed a greater impairment of the [Ca^2+^]_c_ responses when BAPTA was added (Fig 4C and 4D) in comparison to the TFP pretreatment (Fig 4E and 4F).

## Discussion

Calcium is an extraordinarily dynamic and versatile signal molecule [17] that acts as a second messenger in regulating diverse processes in filamentous fungi. These processes include: spore germination, hyphal growth and orientation, sporulation, antifungal drug resistance and pathogenicity [18,44,47–51].

The bioluminescent Ca^2+^ reporter aequorin has proven a useful methodology in filamentous fungi for routine measurements of [Ca^2+^]_c_ dynamics in living cells at the cell population level in response to different stimuli, stresses, antifungal drugs and peptides [23,31,41,43,52–55]. Our calibration gave no indication that the aequorin in our aequorin-expressing strains is measuring [Ca^2+^]_c_ concentrations other than those of the cytosol. The resting (unstimulated) level of [Ca^2+^]_c_ measured was 0.05–0.1 μM which undoubtedly does not correspond to the high free Ca^2+^ concentration present in organelles such as vacuoles, reported to be roughly 2.5 mM, which is 25,000-fold higher than the concentration in the cytosol (46). Moreover, no targeting sequence was added to the aequorin gene and thus we expect the aequorin to be exclusively cytosolic. In this study, an *A*. *fumigatus* aequorin-expressing strain was constructed by modification of the clinical isolate CBS 144–89 (CEA10), thereby achieving a targeted and single integration of the aequorin gene into the *A*. *fumigatus* genome (S1 Fig). In order to promote future mutational analyses of Ca^2+^-signalling we also generated an *A*. *fumigatus* aequorin-expressing strain in a *ΔakuB* ^KU80^ background (S2 Fig). The *ΔakuB* ^KU80^ strain has been extensively used for the generation of mutants in *A*. *fumigatus* because targeted recombination of exogenous DNA is facilitated by an increased homologous recombination frequency. This is due to the absence of the *akuB* gene (AFUA_2G02620), the product of which governs the rejoining of non-homologous DNA fragments. The resultant AEQ^*ΔakuB*^ strain provides a recipient reporter strain, in which targeted deletion of desired genes can be easily achieved using standard molecular techniques and repair of pyrimidine auxotrophy. This auxotrophic recipient achieves similar growth to prototrophic isolates when supplemented with uridine and uracil (50 mM and 25 mM, respectively).

We next assessed the utility of the isolates as reporters of [Ca^2+^]_c_ dynamics by challenging the cells with an array of different stressors, likely to be encountered by *A*. *fumigatus* conidial germlings in the environment and in the mammalian lungs during infection. The Ca^2+^ signatures associated with each physico-chemical treatment was found to be distinct, suggesting the involvement of a different combination of Ca^2+^-signalling machinery components in each stress response (Figs 3 and 4). Furthermore, the conidial germlings of the pathogen produced dose- and growth-dependent Ca^2+^-signatures in response to each type of stressor (Fig 3). Interestingly in the case of exposure to human serum which contains ~ 2 mM extracellular Ca^2+^, the signature observed was different and greater than the that recorded by supplementing the media with 5 mM Ca^2+^ only, thereby indicating that other factors apart from Ca^2+^ in the serum contribute to the Ca^2+^-signature.

Previously, we have reported that conidial germlings and vegetative hyphae of *N*. *crassa*, *A*. *awamori* and *A*. *niger* also respond to mechanical perturbation, hypo-osmotic shock and high external Ca^2+^ [23,41,52,56] by producing distinctly different Ca^2+^-signatures. However, the [Ca^2+^]_c_ values reported in these studies were low and inaccurate. The [Ca^2+^]_c_ values reported in the present study, and a previous one on *N*. *crassa* [43], are much more precise as the calibration method to convert RLUs into [Ca^2+^]_c_ concentrations using the aequorin reporter has been significantly improved [39]. In the present investigation, *A*. *fumigatus* germlings were found to exhibit a [Ca^2+^]_c_ resting level of 0.05–0.1 μM and to undergo transient [Ca^2+^]_c_ changes with amplitudes that ranged from 0.15 up to 0.9 μM depending on the stress applied.

Application of both the Ca^2+^-chelator BAPTA and the calmodulin antagonist TFP, both of which have been previously used with filamentous fungi [52,57], significantly impacted observed Ca^2+-^signatures. The chelation of external Ca^2+^ with BAPTA resulted in a significant reduction of the maximal [Ca^2+^]_c_ amplitude in response to hypo-osmotic shock, hyper-osmotic shock, oxidative stress and 50% human serum (Fig 4C). Thus the [Ca^2+^]_c_ responses to these environmental challenges are likely to involve influx of extracellular Ca^2+^. TFP reduced the maximal [Ca^2+^]_c_ amplitude in response to high extracellular Ca^2+^ and oxidative stress suggesting that Ca^2+^-influx is mediated by calmodulin in response to these treatments (Fig 4E).

Taken together, our results show that *A*. *fumigatus* responds to specific stressors via transient increases in [Ca^2+^]_c_ exhibiting stress-specific signatures. Extracellular Ca^2+^ and the primary intracellular Ca^2+^-receptor calmodulin play different roles in generating these specific Ca^2+^-signatures and in regulating [Ca^2+^]_c_ homeostasis. How the biochemical machinery of the fungal cell is able to decode these Ca^2+^-signatures and convert them into specific cellular responses is thus far unclear. However, certain types of environmental stress (e.g. high extracellular Ca^2+^) result in the recruitment of the transcription factor, CrzA, to nuclei, which up- and down-regulates a range of genes which support stress adaptation (Loss and Bertuzzi *et al*., submitted,[28,58]. In our study, exposure to the stressors tested of the fungus grown on solid medium in Petri dish cultures (Fig 5A) or in liquid medium in multiwell plates (Fig 5B) resulted in significant growth inhibition, with the exception of human serum, which stimulated growth, possibly as a result of increasing the nutrient status of the medium (Fig 5). However, perturbation of growth in response to the same challenges was found to be only dependent on extracellular Ca^2+^ for oxidative stress and the addition of human serum.

Upon challenge with H_2_O_2_, oxidative stress was found to prompt a Ca^2+^-signature dependent upon entry of extracellular Ca^2+^ into *A*. *fumigatus* cells (Fig 4A). Accordingly, BAPTA-mediated chelation of extracellular Ca^2+^ significantly reduced the maximal [Ca^2+^]_c_ amplitude (Fig 4C and S9 Fig). TFP treatment prompted a comparable reduction in maximal [Ca^2+^]_c_ amplitude, but also significantly elevated the post-stimulatory [Ca^2+^]_c_ resting level (Fig 4E and S9 Fig), suggesting a role for calmodulin in mediating both Ca^2+^-influx and the return of the [Ca^2+^]_c_ concentration to its normal resting level following exposure to oxidative stress. Oxidative stress, imposed by the addition of 2.5 mM H_2_O_2_, was the only challenge found to significantly reduce fungal growth (Fig 5), an effect which was significantly potentiated by BAPTA-mediated chelation of extracellular Ca^2+^ (Fig 5B). Taken together, these data suggest that *A*. *fumigatus* is highly sensitive to oxidative stress, and that tolerance of oxidative stress requires Ca^2+-^mediated signalling. Concordant with this hypothesis, *A*. *fumigatus* null mutants *ΔcchA*, *ΔmidA*, and *ΔcchAΔmidA* have been found to be sensitive to paraquat, an oxidative stress inducer [25].

In the pathogenic yeast *Candida albicans*, a similar mechanistic basis for oxidative stress tolerance is suggested by heightened sensitivity, relative to untreated cells, of *C*. *albicans* to H_2_O_2_ in the presence of BAPTA, or the L-type voltage-gated Ca^2+^-channel blocker verapamil [59,60]. In addition, *C*. *albicans* mutants lacking *CCH1*, *MID1* or *ECM7* (involved in [Ca^2+^]_c_ homeostasis) show increased sensitivity to oxidative stress caused by H_2_O_2_ or menadione, and decreased expression of oxidative stress response genes, such as *GLR1* (glutathione reductase), *TRR1* (thioredoxin reductase) and *IPF7817* (NAD(P)H oxidoreductase family protein). In *Schizosaccharomyces pombe*, deletion of c*mk2*, which encodes a serine-threonine kinase related to Ca^2+^/calmodulin-dependent kinases, results in hypersensitivity to oxidative stress [61]. Similarly, growth of a *C*. *albicans cmk1Δ/Δcmk2Δ* mutant is severely inhibited by H_2_O_2_ treatment [62]. In light of the dependency of *A*. *fumigatus* upon extracellular Ca^2+^ for growth and stress tolerance, and as previously proposed by various authors [18,23,63,64], the Ca^2+^ signalling machinery is a plausible target for the development of novel antifungal therapies. In this work we have developed the tools to measure and analyse [Ca^2+^]_c_, and have demonstrated correlations between external stimuli, specific changes in [Ca^2+^]_c_ and either the inhibition or stimulation of growth. The mechanistic basis of how specific [Ca^2+^]_c_ signals are generated, and converted into specific responses, in filamentous fungi remains to be addressed. The recent development of genetically encoded Ca^2+^-sensitive probes that can be used to image subcellular [Ca^2+^]_c_ dynamics in filamentous fungi [51] will play critical roles in furthering our understanding of Ca^2+^-signalling in filamentous fungi. Such studies, already underway in our laboratories, will also fuel the development of improved antifungal therapies against deadly fungal pathogens.

## Supporting Information

S1 Data Sequence of plasmids constructed in this study. (PDF)

S1 Fig Design and construction of the *A*. *fumigatus* reporter strain AEQ^CEA10^. (A) pAEQ(I) directs the expression of the *aeqS* gene under the control of the constitutive *A*. *nidulans* promoter *gpdA* ^*P*^. (B) Targeted genomic integration of circular pAEQ(I) is directed by *his2A* ^*t*^. (C) Single, targeted integration of the aequorin expression construct in AEQ^CEA10^, as verified by Southern blotting using an *aeqS*-specific probe. (D) Southern blotting strategy. No band is expected for the wild type isolate, whereas a single band, ranging from 1865 to 3862 bp, dependent upon the precise site of integration, is expected for the reporter strain.
(PDF)

S2 Fig Design and construction of the *A*. *fumigatus* reporter strain AEQ^*ΔakuB*^. (A) In pAEQ(II), the *gpdA* ^*P*^-*aeqS* expression cassette is sandwiched between 1 kb, 5’ and 3’ flanking regions of the *KU80* gene (AFU_2G02620). (B) Strategy for targeting of the *ku80* ^3’^-*aeqS*-*ku80* ^5’^ cassette to the *KU80* genetic locus. (C) Single, targeted integration verified by Southern blotting using an *aeqS*-specific probe. (D) Southern blotting strategy. No band is expected for the wild type isolate, whereas a single band of 13576 bp is expected for the reporter strain.
(PDF)

S3 Fig Aequorin expression in the reporter strains. *A*. *fumigatus* protein extracts were prepared after growth of the strains at 25°C for 16 or 20 h. Western blotting analysis of *A*. *fumigatus* protein extracts demonstrates robust expression of recombinant aequorin protein in both AEQ^CEA10^ and AEQ^*ΔakuB*^ isolates. Aequorin was detected using a polyclonal rabbit anti-aequorin antibody (Abcam).
(PDF)

S4 Fig Comparison of spore germination efficiency in aequorin expressing strains AEQ^CEA10^ and AEQ^*ΔakuB*^ and progenitor isolates CEA10 and *ΔakuB* ^KU80^. Percentages of ungerminated and germinated conidia were microscopically quantified at different times (5 h to 11.5 h) whilst incubated in AMM at 37°C.
(PDF)

S5 Fig Phenotypic analysis of the aequorin expressing strains. (A) Microscopic visualization of germinated conidia and germlings of the parental and transformant strains after 11.5 h of incubation at 37°C. (B) Radial growth phenotype on solid agar plates after 48 h of incubation at 37°C and (C) colony diameter measurements (in mm). Bar: 10 μm.
(PDF)

S6 Fig Data shown in Fig 2 with error bars shown. (PDF)

S7 Fig Ca^2+^-signatures in response to mechanical perturbation and hypo-osmotic shock in the aequorin expressing strain AEQ^*ΔakuB*^ are similar to those of the AEQ^CEA10^ strain. Each stressor was applied at two time-points of growth (24 and 28 h) at 25°C. Cultures were also microscopically analysed in order to compare the stage of conidial germination and germ tube growth with the [Ca^2+^]_c_ response. Average values ± SD error for six technical replicates are shown. The arrows indicate the point at which each stress was applied via the injectors of the plate reader. Bar: 10 μm.
(PDF)

S8 Fig Data shown in Fig 3 with error bars shown. (PDF)

S9 Fig Impact of the Ca^2+^-modulators BAPTA and TFP on the transient [Ca^2+^]_c_ signature caused by the exposure to stresses. *A*. *fumigatus* AEQ^CEA10^ cultures were pre-treated for 30 min with either 5 mM BAPTA or 50 μM TFP prior to challenge with stressors (applied at points indicated by arrows). Note the influence of the modulators on the [Ca^2+^]_c_ amplitudes and post-stimulatory [Ca^2+^]_c_ resting levels. Statistical comparisons between untreated and treated samples are presented in Fig 4.
(PDF)

S1 Table Effect of the available aequorin on the [Ca^2+^]_c_ resting level. (PDF)

S2 Table Excel spreadsheet for the conversion of RLUs into [Ca^2+^]_c_. (XLSX)

S3 Table Exemplar dataset (mechanical perturbation) and conversion of RLUs into [Ca^2+^]_c_. (XLSX)

S4 Table Raw data for timescale of germination for *A*. *fumigatus* strains used in this study. (XLSX)

## References

[1] Casadevall A, Pirofski LA (2003) The damage-response framework of microbial pathogenesis. Nat Rev Microbiol 1: 17–24. 15040176 10.1038/nrmicro732 PMC7097162

[2] Askew DS (2008) *Aspergillus fumigatus*: virulence genes in a street-smart mold. Curr Opin Microbiol 11: 331–337. 10.1016/j.mib.2008.05.009 18579432 PMC2559812

[3] McCormick A, Loeffler J, Ebel F (2010) *Aspergillus fumigatus*: contours of an opportunistic human pathogen. Cell Microbiol 12: 1535–1543. 10.1111/j.1462-5822.2010.01517.x 20716206

[4] Balloy V, Chignard M (2009) The innate immune response to *Aspergillus fumigatus* . Microbes Infect 11: 919–927. 10.1016/j.micinf.2009.07.002 19615460

[5] Baddley JW, Andes DR, Marr KA, Kontoyiannis DP, Alexander BD, et al (2010) Factors associated with mortality in transplant patients with invasive aspergillosis. Clin Infect Dis 50: 1559–1567. 10.1086/652768 20450350 PMC2874071

[6] Kontoyiannis DP, Marr KA, Park BJ, Alexander BD, Anaissie EJ, et al (2010) Prospective surveillance for invasive fungal infections in hematopoietic stem cell transplant recipients, 2001–2006: overview of the Transplant-Associated Infection Surveillance Network (TRANSNET) Database. Clin Infect Dis 50: 1091–1100. 10.1086/651263 20218877

[7] Pappas PG, Alexander BD, Andes DR, Hadley S, Kauffman CA, et al (2010) Invasive fungal infections among organ transplant recipients: results of the Transplant-Associated Infection Surveillance Network (TRANSNET). Clin Infect Dis 50: 1101–1111. 10.1086/651262 20218876

[8] Brown GD, Denning DW, Gow NA, Levitz SM, Netea MG, et al (2012) Hidden killers: human fungal infections. Sci Transl Med 4: 165rv113. 10.1126/scitranslmed.3004404 23253612

[9] Kleinkauf N, Verweij P, Arendrup M, Donnelly P, Cuenca-Estrella M, et al (2013) Risk assessment on the impact of environmental usage of triazoles on the development and spread of resistance to medical triazoles in Aspergillus species. Stockholm: European Centre for Disease Control.

[10] Latge JP (1999) *Aspergillus fumigatus* and aspergillosis. Clin Microbiol Rev 12: 310–350. 10194462 10.1128/cmr.12.2.310 PMC88920

[11] Bains SN, Judson MA (2012) Allergic bronchopulmonary aspergillosis. Clin Chest Med 33: 265–281. 10.1016/j.ccm.2012.02.003 22640845

[12] Denning DW, Pleuvry A, Cole DC (2013) Global burden of allergic bronchopulmonary aspergillosis with asthma and its complication chronic pulmonary aspergillosis in adults. Med Mycol 51: 361–370. 10.3109/13693786.2012.738312 23210682

[13] Denning DW, Pleuvry A, Cole DC (2013) Global burden of chronic pulmonary aspergillosis complicating sarcoidosis. Eur Respir J 41: 621–626. 10.1183/09031936.00226911 22743676

[14] Denning DW, Pleuvry A, Cole DC (2011) Global burden of chronic pulmonary aspergillosis as a sequel to pulmonary tuberculosis. Bull World Health Organ 89: 864–872. 10.2471/BLT.11.089441 22271943 PMC3260898

[15] Ohba R, Furuyama K, Yoshida K, Fujiwara T, Fukuhara N, et al (2013) Clinical and genetic characteristics of congenital sideroblastic anemia: comparison with myelodysplastic syndrome with ring sideroblast (MDS-RS). Ann Hematol 92: 1–9. 10.1007/s00277-012-1564-5 22983749 PMC3536986

[16] Henderson AG, Ehre C, Button B, Abdullah LH, Cai LH, et al (2014) Cystic fibrosis airway secretions exhibit mucin hyperconcentration and increased osmotic pressure. J Clin Invest 124: 3047–3060. 10.1172/JCI73469 24892808 PMC4072023

[17] Clapham DE (2007) Calcium signaling. Cell 131: 1047–1058. 18083096 10.1016/j.cell.2007.11.028

[18] Steinbach WJ, Cramer RA Jr., Perfect BZ, Asfaw YG, Sauer TC, et al (2006) Calcineurin controls growth, morphology, and pathogenicity in *Aspergillus fumigatus* . Eukaryot Cell 5: 1091–1103. 16835453 10.1128/EC.00139-06 PMC1489296

[19] da Silva Ferreira ME, Heinekamp T, Hartl A, Brakhage AA, Semighini CP, et al (2007) Functional characterization of the *Aspergillus fumigatus* calcineurin. Fungal Genet Biol 44: 219–230. 16990036 10.1016/j.fgb.2006.08.004

[20] Juvvadi PR, Fortwendel JR, Rogg LE, Burns KA, Randell SH, et al (2011) Localization and activity of the calcineurin catalytic and regulatory subunit complex at the septum is essential for hyphal elongation and proper septation in *Aspergillus fumigatus* . Mol Microbiol 82: 1235–1259. 10.1111/j.1365-2958.2011.07886.x 22066998 PMC3225650

[21] Meyer V, Arentshorst M, Flitter SJ, Nitsche BM, Kwon MJ, et al (2009) Reconstruction of signaling networks regulating fungal morphogenesis by transcriptomics. Eukaryot Cell 8: 1677–1691. 10.1128/EC.00050-09 19749177 PMC2772408

[22] Greene V, Cao H, Schanne FA, Bartelt DC (2002) Oxidative stress-induced calcium signalling in *Aspergillus nidulans* . Cell Signal 14: 437–443. 11882388 10.1016/s0898-6568(01)00266-2

[23] Nelson G, Kozlova-Zwinderman O, Collis AJ, Knight MR, Fincham JR, et al (2004) Calcium measurement in living filamentous fungi expressing codon-optimized aequorin. Mol Microbiol 52: 1437–1450. 15165245 10.1111/j.1365-2958.2004.04066.x

[24] Warwar V, Dickman MB (1996) Effects of Calcium and Calmodulin on Spore Germination and Appressorium Development in *Colletotrichum trifolii* . Appl Environ Microbiol 62: 74–79. 16535223 10.1128/aem.62.1.74-79.1996 PMC1388743

[25] de Castro PA, Chiaratto J, Winkelstroter LK, Bom VL, Ramalho LN, et al (2014) The involvement of the Mid1/Cch1/Yvc1 calcium channels in *Aspergillus fumigatus* virulence. PLoS One 9: e103957 10.1371/journal.pone.0103957 25083783 PMC4118995

[26] Dinamarco TM, Freitas FZ, Almeida RS, Brown NA, dos Reis TF, et al (2012) Functional characterization of an *Aspergillus fumigatus* calcium transporter (PmcA) that is essential for fungal infection. PLoS One 7: e37591 10.1371/journal.pone.0037591 22649543 PMC3359301

[27] Cramer RA Jr., Perfect BZ, Pinchai N, Park S, Perlin DS, et al (2008) Calcineurin target CrzA regulates conidial germination, hyphal growth, and pathogenesis of *Aspergillus fumigatus* . Eukaryot Cell 7: 1085–1097. 10.1128/EC.00086-08 18456861 PMC2446674

[28] Soriani FM, Malavazi I, da Silva Ferreira ME, Savoldi M, Von Zeska Kress MR, et al (2008) Functional characterization of the *Aspergillus fumigatus* CRZ1 homologue, CrzA. Mol Microbiol 67: 1274–1291. 10.1111/j.1365-2958.2008.06122.x 18298443

[29] Steinbach WJ, Cramer RA Jr., Perfect BZ, Henn C, Nielsen K, et al (2007) Calcineurin inhibition or mutation enhances cell wall inhibitors against *Aspergillus fumigatus* . Antimicrob Agents Chemother 51: 2979–2981. 17502415 10.1128/AAC.01394-06 PMC1932494

[30] Fortwendel JR, Juvvadi PR, Pinchai N, Perfect BZ, Alspaugh JA, et al (2009) Differential effects of inhibiting chitin and 1,3-β-D-glucan synthesis in ras and calcineurin mutants of *Aspergillus fumigatus* . Antimicrob Agents Chemother 53: 476–482. 10.1128/AAC.01154-08 19015336 PMC2630655

[31] Bagar T, Bencina M (2012) Antiarrhythmic drug amiodarone displays antifungal activity, induces irregular calcium response and intracellular acidification of *Aspergillus niger*—amiodarone targets calcium and pH homeostasis of *A*. *niger* . Fungal Genet Biol 49: 779–791. 10.1016/j.fgb.2012.07.007 22906851

[32] Pontecorvo G, Roper JA, Hemmons LM, Macdonald KD, Bufton AW (1953) The genetics of *Aspergillus nidulans* . Adv Genet 5: 141–238. 13040135 10.1016/s0065-2660(08)60408-3

[33] Monod M, Paris S, Sarfati J, Jaton-Ogay K, Ave P, et al (1993) Virulence of alkaline protease-deficient mutants of *Aspergillus fumigatus* . FEMS Microbiol Lett 106: 39–46. 8095038 10.1111/j.1574-6968.1993.tb05932.x

[34] da Silva Ferreira ME, Kress MR, Savoldi M, Goldman MH, Hartl A, et al (2006) The akuB(KU80) mutant deficient for nonhomologous end joining is a powerful tool for analyzing pathogenicity in *Aspergillus fumigatus* . Eukaryot Cell 5: 207–211. 16400184 10.1128/EC.5.1.207-211.2006 PMC1360264

[35] Wagener J, Echtenacher B, Rohde M, Kotz A, Krappmann S, et al (2008) The putative α-1,2-mannosyltransferase AfMnt1 of the opportunistic fungal pathogen *Aspergillus fumigatus* is required for cell wall stability and full virulence. Eukaryot Cell 7: 1661–1673. 10.1128/EC.00221-08 18708564 PMC2568062

[36] Szewczyk E, Nayak T, Oakley CE, Edgerton H, Xiong Y, et al (2007) Fusion PCR and gene targeting in *Aspergillus nidulans* . Nat Protocols 1: 3111–3120. 10.1038/nprot.2006.405 17406574

[37] Kubodera T, Yamashita N, Nishimura A (2000) Pyrithiamine resistance gene (*ptrA*) of *Aspergillus oryzae*: cloning, characterization and application as a dominant selectable marker for transformation. Biosci Biotechnol Biochem 64: 1416–1421. 10945258 10.1271/bbb.64.1416

[38] Fernández-Martinez J, Brown C, DI ez E, Tilburn J, Arst H Jr, et al (2003) Overlap of nuclear localisation signal and specific DNA-binding residues within the zinc finger domain of PacC. Journal of molecular biology 334: 667–684. 14636595 10.1016/j.jmb.2003.09.072

[39] Bonora M, Giorgi C, Bononi A, Marchi S, Patergnani S, et al (2013) Subcellular calcium measurements in mammalian cells using jellyfish photoprotein aequorin-based probes. Nat Protoc 8: 2105–2118. 10.1038/nprot.2013.127 24113784

[40] Berridge MJ, Lipp P, Bootman MD (2000) The versatility and universality of calcium signalling. Nat Rev Mol Cell Biol 1: 11–21. 11413485 10.1038/35036035

[41] Bencina M, Legisa M, Read ND (2005) Cross-talk between cAMP and calcium signalling in *Aspergillus niger* . Mol Microbiol 56: 268–281. 15773995 10.1111/j.1365-2958.2005.04541.x

[42] Binder U, Oberparleiter C, Meyer V, Marx F (2010) The antifungal protein PAF interferes with PKC/MPK and cAMP/PKA signalling of *Aspergillus nidulans* . Mol Microbiol 75: 294–307. 10.1111/j.1365-2958.2009.06936.x 19889092 PMC2814085

[43] Munoz A, Chu M, Marris PI, Sagaram US, Kaur J, et al (2014) Specific domains of plant defensins differentially disrupt colony initiation, cell fusion and calcium homeostasis in *Neurospora crassa* . Mol Microbiol 92: 1357–1374. 10.1111/mmi.12634 24773060

[44] Zelter A, Bencina M, Bowman BJ, Yarden O, Read ND (2004) A comparative genomic analysis of the calcium signaling machinery in *Neurospora crassa*, *Magnaporthe grisea*, and *Saccharomyces cerevisiae* . Fungal Genet Biol 41: 827–841. 15288019 10.1016/j.fgb.2004.05.001

[45] Bencina M, Bagar T, Lah L, Krasevec N (2009) A comparative genomic analysis of calcium and proton signaling/homeostasis in Aspergillus species. Fungal Genet Biol 46 Suppl 1: S93–S104. 19610176 10.1016/j.fgb.2008.07.019

[46] Vandonselaar M, Hickie RA, Quail JW, Delbaere LT (1994) Trifluoperazine-induced conformational change in Ca^2+^-calmodulin. Nat Struct Biol 1: 795–801. 7634090 10.1038/nsb1194-795

[47] Bowman BJ, Abreu S, Margolles-Clark E, Draskovic M, Bowman EJ (2011) Role of four calcium transport proteins, encoded by nca-1, nca-2, nca-3, and cax, in maintaining intracellular calcium levels in *Neurospora crassa* . Eukaryot Cell 10: 654–661. 10.1128/EC.00239-10 21335528 PMC3127652

[48] Brand A, Shanks S, Duncan VM, Yang M, Mackenzie K, et al (2007) Hyphal orientation of *Candida albicans* is regulated by a calcium-dependent mechanism. Curr Biol 17: 347–352. 17275302 10.1016/j.cub.2006.12.043 PMC1885950

[49] Cavinder B, Trail F (2012) Role of Fig1, a component of the low-affinity calcium uptake system, in growth and sexual development of filamentous fungi. Eukaryot Cell 11: 978–988. 10.1128/EC.00007-12 22635922 PMC3416067

[50] Kim YK, Li D, Kolattukudy PE (1998) Induction of Ca^2+^-calmodulin signaling by hard-surface contact primes *Colletotrichum gloeosporioides* conidia to germinate and form appressoria. J Bacteriol 180: 5144–5150. 9748448 10.1128/jb.180.19.5144-5150.1998 PMC107551

[51] Kim HS, Czymmek KJ, Patel A, Modla S, Nohe A, et al (2012) Expression of the Cameleon calcium biosensor in fungi reveals distinct Ca^2+^ signatures associated with polarized growth, development, and pathogenesis. Fungal Genet Biol 49: 589–601. 10.1016/j.fgb.2012.05.011 22683653

[52] Binder U, Chu M, Read ND, Marx F (2010) The antifungal activity of the Penicillium chrysogenum protein PAF disrupts calcium homeostasis in *Neurospora crassa* . Eukaryot Cell 9: 1374–1382. 10.1128/EC.00050-10 20622001 PMC2937333

[53] Binder U, Bencina M, Eigentler A, Meyer V, Marx F (2011) The *Aspergillus giganteus* antifungal protein AFPNN5353 activates the cell wall integrity pathway and perturbs calcium homeostasis. BMC Microbiol 11: 209 10.1186/1471-2180-11-209 21943024 PMC3197501

[54] Gonçalves AP, Cordeiro JM, Monteiro J, Muñoz A, Correia-de-Sá P, et al (2014) Activation of a TRP-like channel and intracellular calcium dynamics during phospholipase C-mediated cell death. Journal of Cell Science. 10.1242/jcs.152058 PMC4150065 25037570

[55] Munoz A, Marcos JF, Read ND (2012) Concentration-dependent mechanisms of cell penetration and killing by the *de novo* designed antifungal hexapeptide PAF26. Mol Microbiol 85: 89–106. 10.1111/j.1365-2958.2012.08091.x 22646057

[56] Troppens DM, Chu M, Holcombe LJ, Gleeson O, O'Gara F, et al (2013) The bacterial secondary metabolite 2,4-diacetylphloroglucinol impairs mitochondrial function and affects calcium homeostasis in *Neurospora crassa* . Fungal Genet Biol 56: 135–146. 10.1016/j.fgb.2013.04.006 23624246

[57] de Carvalho MJ, Amorim Jesuino RS, Daher BS, Silva-Pereira I, de Freitas SM, et al (2003) Functional and genetic characterization of calmodulin from the dimorphic and pathogenic fungus *Paracoccidioides brasiliensis* . Fungal Genet Biol 39: 204–210. 12892633 10.1016/s1087-1845(03)00044-6

[58] Soriani F, Malavazi I, Savoldi M, Espeso E, Dinamarco T, et al (2010) Identification of possible targets of the *Aspergillus fumigatus* CRZ1 homologue, CrzA. BMC Microbiology 10: 12 10.1186/1471-2180-10-12 20078882 PMC2818617

[59] Yu Q, Xiao C, Zhang K, Jia C, Ding X, et al (2014) The calcium channel blocker verapamil inhibits oxidative stress response in *Candida albicans* . Mycopathologia 177: 167–177. 10.1007/s11046-014-9735-7 24577794

[60] Teng J, Goto R, Iida K, Kojima I, Iida H (2008) Ion-channel blocker sensitivity of voltage-gated calcium-channel homologue Cch1 in *Saccharomyces cerevisiae* . Microbiology 154: 3775–3781. 10.1099/mic.0.2008/021089-0 19047745

[61] Sanchez-Piris M, Posas F, Alemany V, Winge I, Hidalgo E, et al (2002) The serine/threonine kinase Cmk2 is required for oxidative stress response in fission yeast. J Biol Chem 277: 17722–17727. 11886858 10.1074/jbc.M200104200

[62] Ding X, Yu Q, Zhang B, Xu N, Jia C, et al (2014) The type II Ca2+/calmodulin-dependent protein kinases are involved in the regulation of cell wall integrity and oxidative stress response in Candida albicans. Biochem Biophys Res Commun 446: 1073–1078. 10.1016/j.bbrc.2014.03.059 24661877

[63] Juvvadi PR, Lamoth F, Steinbach WJ (2014) Calcineurin as a multifunctional regulator: unraveling novel functions in fungal stress responses, hyphal growth, drug resistance, and pathogenesis. Fungal Biol Rev 28: 56–69. 25383089 10.1016/j.fbr.2014.02.004 PMC4219591

[64] Liu S, Hou Y, Liu W, Lu C, Wang W, et al (2015) Components of the calcium-calcineurin signaling pathway in fungal cells and their potential as antifungal targets. Eukaryot Cell 14: 324–334. 10.1128/EC.00271-14 25636321 PMC4385803

