# Supplementary material for: Different Stress-Induced Calcium Signatures Are Reported by Aequorin-Mediated Calcium Measurements in Living Cells of Aspergillus fumigatus
Source: PLoS One. 2015 Sep 24;10(9):e0138008. doi: 10.1371/journal.pone.0138008 (PMC4581630; doi:10.1371/journal.pone.0138008)
Supplement: S1 Data — (PDF) [file pone.0138008.s001.pdf]

SUPPLEMENTARY DATA 1. Sequence of plasmids constructed in this study.

pAEQ(I)

In red the half sequence of the PmeI site after the cloning

PmeI

CAGACATCACCGTTTATGACCTCCAAGCAGTACTCCGTCAAGCTTACCTCCGACTTCGACAA

CCCCCGCTGGATCGGCCGCCACAAGCACATGTTCAACTTCCTCGACGTCAACCACAACGGCA

AGATTTCCCTCGACGAGATGGTCTACAAGGCCTCCGACATCGTCATCAACAACCTCGGCGCT

ACCCCCGAGCAGGCCAAGCGCCACAAGGACGCCGTCGAGGCCTTCTTCGGCGGTGCCGGCAT

GAAGTACGGCGTCGAGACCGACTGGCCCGCCTACATCGAGGGCTGGAAGAAGCTCGCCACCG

ACGAGCTCGAGAAGTACGCCAAGAACGAGCCCACCCTCATCCGCATCTGGGGCGACGCCCTC

TTCGACATCGTCGACAAGGACCAGAACGGTGCCATCACCCTCGACGAGTGGAAGGCCTACAC

CAAGGCCGCCGGCATCATTCAGTCCAGCGAGGACTGCGAAGAGACCTTCCGCGTCTGCGACA

TCGACGAGTCCGGCCAGCTCGATGTCGATGAGATGACCCGCCAGCACCTCGGCTTCTGGTAC

ACCATGGACCCCGCCTGCGAGAAGCTCTACGGCGGTGCCGTCCCCTAAAAACGCCATGTCTA

PmeI

pAEQ(II)

In red the half sequence of the SfiI restriction site

SfiI *ku80*$^{5'}$

TCGAGCTCGGTACGGCCATATAGGCCCATGAAGGCGCATCTGCACGATGCGCGACTCCATCT

CACAAGACGGGACCAGGAAACGACGCCGCAAAAGCAGCATGAGAGCTCTGCGCGGGCGAAGA

GCAGTCTTGTACGAGCTGGAAGTCTGATCGGGGCGCTGGAGGACGGCCTGAAGAATCTTGGG

GATGCCAATGCGGATGCGAGGTCCACTGCGTCTGGGAAGAGGGGTGCTTGGGGGAGCGGAAA

TGGGCTGGGCGACGGGGAGATTCGACGAAGAAAGGACTTGCTGGTTAATGCGAAGAAGGAGA

AGAATGGATTGGAGGATTTGTTAAACGCCATGGCTGCTAAGAGCAGGATTGATAATGCGGTT

GCGTCGATACAAGACAAGGAGGCGTTGGTTGGGTCTGCGAGCCGGAAGCCACCTCGTTCGGG

GAGGGTGCTGGGCAAGGAGACGGAGCGGACTCGCGAGCTAGATAACCAAGGCGTTTTGCAGT
TACAGAAGCGGACGATGGAGGATCAAGATATGAGCATTGAAGAGTTGAGGAAGATTGTACAA
CGCCAGAAGGAACTGGGGATTGCTATCAATGCTGAGTTGGAGATTCAGAATGAGCTTCTGAA
GCTCACGGATGAAGATACGGACAGGTATGTGTGGACCCAGGCTTCGGCTCTAATAACCCATT
CTAACATCTGTCGCCTAAAGGTTAGGGAAGAAGATCGAAATCGGGAACAAGAGGGTTGGCAA
AATATCTTAGCTTGGCATCTCACGGGCACTTCTGCTTCTACACAGCTTATTCATTGTTTCTG

gpdA^P

AACCATTTAGAGGAGCGACT**ATCTTTGCCCGGTGTATGAAACCGGAAAGGCCGCTCAGGAGC**
TGGCCAGCGGCGCAGACCGGGAACACAAGCTGGCAGTCGACCCATCCGGTGCTCTGCACTCG
ACCTGCTGAGGTCCCTCAGTCCCTGGTAGGCAGCTTTGCCCCGTCTGTCCGCCCGGTGTGTC
GGCGGGGTTGACAAGGTCGTTGCGTCAGTCCAACATTTGTTGCCATATTTTCCTGCTCTCCC
CACCAGCTGCTCTTTTCTTTTCTCTTTCTTTTCCCATCTTCAGTATATTCATCTTCCCATCC
AAGAACCTTTATTTCCCCTAAGTAAGTACTTTGCTACATCCATACTCCATCCTTCCCATCCC
TTATTCCTTTGAACCTTTCAGTTCGAGCTTTCCCACTTCATCGCAGCTTGACTAACAGCTAC

aeqS

CCCGCTTGAGCAGACATCACCGTTTATGACCTCCAAGCAGTACTCCGTCAAGCTTACCTCCG
ACTTCGACAACCCCCGCTGGATCGGCCGCCACAAGCACATGTTCAACTTCCTCGACGTCAAC
CACAACGGCAAGATTTCCCTCGACGAGATGGTCTACAAGGCCTCCGACATCGTCATCAACAA
CCTCGGCGCTACCCCCGAGCAGGCCAAGCGCCACAAGGACGCCGTCGAGGCCTTCTTCGGCG
GTGCCGGCATGAAGTACGGCGTCGAGACCGACTGGCCCGCCTACATCGAGGGCTGGAAGAAG
CTCGCCACCGACGAGCTCGAGAAGTACGCCAAGAACGAGCCCACCCTCATCCGCATCTGGGG
CGACGCCCTCTTCGACATCGTCGACAAGGACCAGAACGGTGCCATCACCCTCGACGAGTGGA
AGGCCTACACCAAGGCCGCCGGCATCATTCAGTCCAGCGAGGACTGCGAAGAGACCTTCCGC
GTCTGCGACATCGACGAGTCCGGCCAGCTCGATGTCGATGAGATGACCCGCCAGCACCTCGG

ku80$^{3'}$

CTTCTGGTACACCATGGACCCCGCCTGCGAGAAGCTCTACGGCGGTGCCGTCCCCTAAGATC

AAGATGCTCTAGAATAGAAATTGAGTCAGGGCATGAGGGAAGGAAGAGCCAGATAAAGGTAG

CTCATTGATGCATGGACAAACACGACTCTCCAGGCAGACGCGCAATATTTCCCGATTGGGTG

TTAGGCAGAACGCGAAGGCACATGACATCATTAATGGAAGGTGCGTGGACATTGCACGTAGT

AAGTGTACATACTTCGGGTCCAATGAAAAGCCGTTGCACCTGTCATATTCCGCAGGACGGGT

GCGTCGAAATATGTTCCGGCATTCCCTAGCTCTTCATCTTCCAACAACCATCTGATCCCGTC

TTGTCGTCTTATCTGCATCCGAATATCCCAGACCCAGCAGTTGACAGATTATAAAGTGCATC

ATTAATTGCTGGACAGGCAATATGACTGATCCTCGCCCCTACCATCTTCTTAGGTACAGAAT

CCCTGGTTATCAGGATGTGAACATGTCGCTGACTTCGGGCGCAAGCTACGGTACCCTATTGG

GTGTGCAAGTGTCCCAGGTGAACTTCATTCTGCCAGCTAGATATCCACTACGCTGTTAACTC

AAAGGCGCAACAGACATTTATTGGGGGCGTTATTGCTTTCCGAGTGCTCCCTCGTCCTCAGT

TCTCAGCGTTGCAAACTTCGATATTCCCGGTGTACTTCACGCTTCAGTCCGCACTCCCCGTA

GTTATCGCCTTGACTTCCAGCAAAGGTGGCCAGCTCAATGGCATTTCCGGACTTCTCGCTCC

SfiI

TGAAAGTCGATTTGGGACACTCCTGCCTCTCGCAACTGTTGCAGTGTCTGGGGGCCTATATG

GCCGGCATGCAAGCTTG
