# Supplementary figures and images for: Different Stress-Induced Calcium Signatures Are Reported by Aequorin-Mediated Calcium Measurements in Living Cells of Aspergillus fumigatus
Source: PLoS One. 2015 Sep 24;10(9):e0138008. doi: 10.1371/journal.pone.0138008 (PMC4581630; doi:10.1371/journal.pone.0138008)

S1 Fig.

**A**

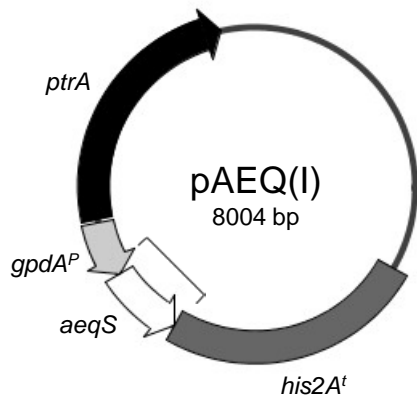

**B**

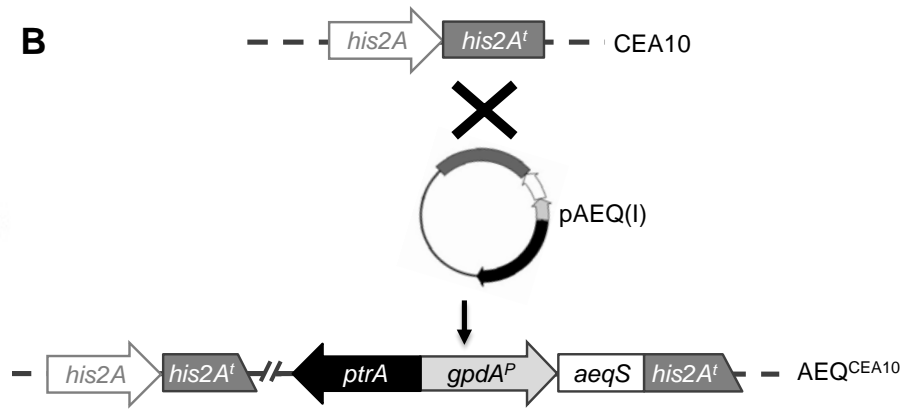

**C**

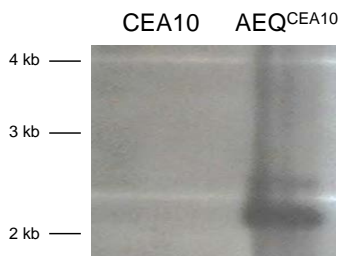

**D**

*A. fumigatus* CEA10

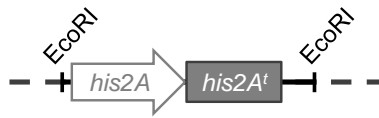

*A. fumigatus* AEQ<sup>CEA10</sup>

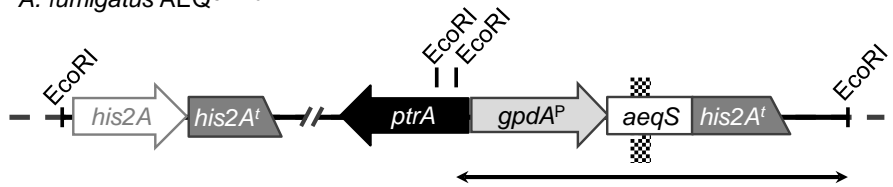

Supplement: S1 Fig — (A) pAEQ(I) directs the expression of the aeqS gene under the control of the constitutive A. nidulans promoter gpdA P. (B) Targeted genomic integration of circular pAEQ(I) is directed by his2A t. (C) Single, targeted integration of the aequorin expression construct in AEQCEA10, as verified by Southern blotting using an aeqS-specific probe. (D) Southern blotting strategy. No band is expected for the wild type isolate, whereas a single band, ranging from 1865 to 3862 bp, dependent upon the precise site of integration, is expected for the reporter strain. (PDF) [file pone.0138008.s002.pdf]

S2 Fig.

A

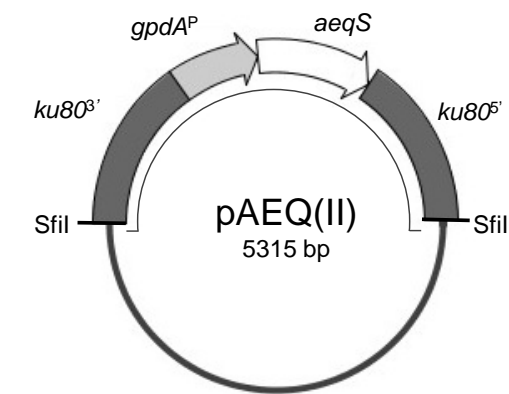

B

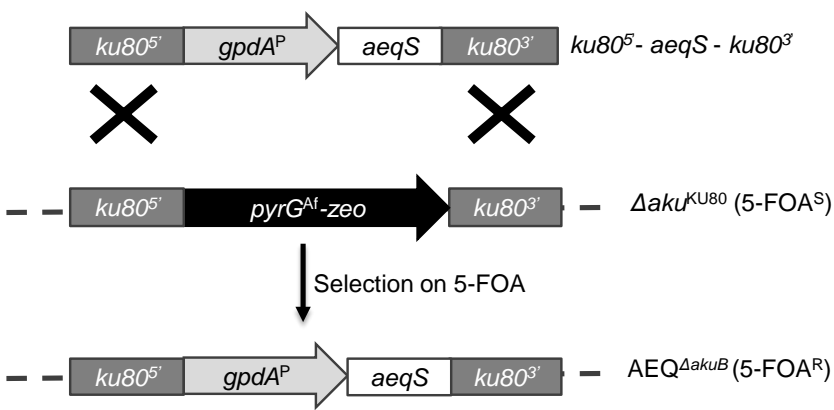

C

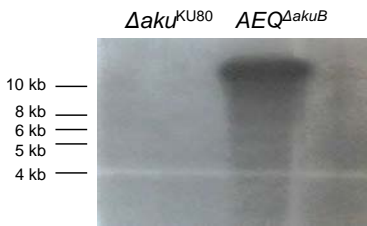

D

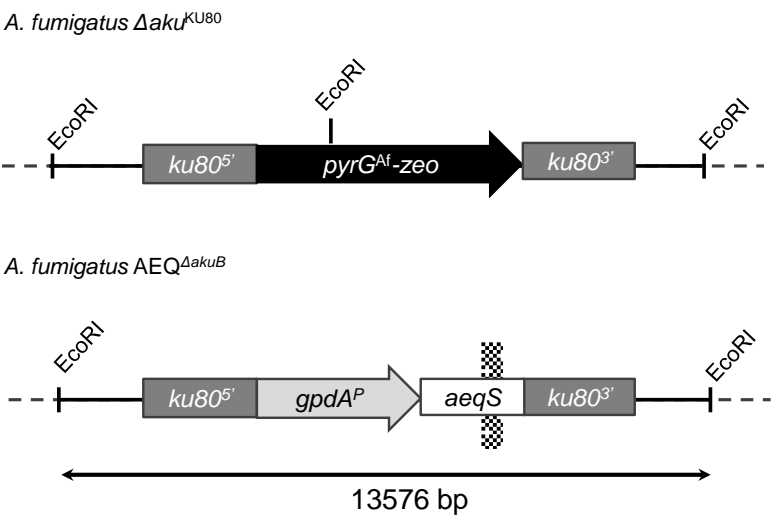

Supplement: S2 Fig — (A) In pAEQ(II), the gpdA P-aeqS expression cassette is sandwiched between 1 kb, 5’ and 3’ flanking regions of the KU80 gene (AFU_2G02620). (B) Strategy for targeting of the ku80 3’-aeqS-ku80 5’ cassette to the KU80 genetic locus. (C) Single, targeted integration verified by Southern blotting using an aeqS-specific probe. (D) Southern blotting strategy. No band is expected for the wild type isolate, whereas a single band of 13576 bp is expected for the reporter strain. (PDF) [file pone.0138008.s003.pdf]

S3 Fig.

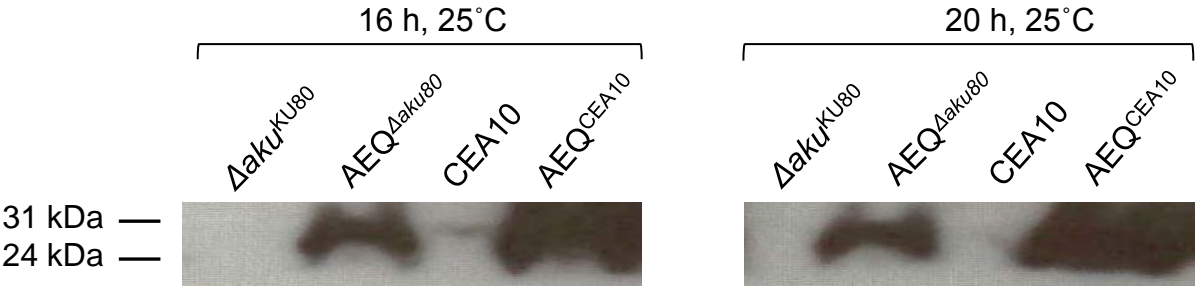

Supplement: S3 Fig — A. fumigatus protein extracts were prepared after growth of the strains at 25°C for 16 or 20 h. Western blotting analysis of A. fumigatus protein extracts demonstrates robust expression of recombinant aequorin protein in both AEQCEA10 and AEQΔakuB isolates. Aequorin was detected using a polyclonal rabbit anti-aequorin antibody (Abcam). (PDF) [file pone.0138008.s004.pdf]

S4 Fig.

CEA10

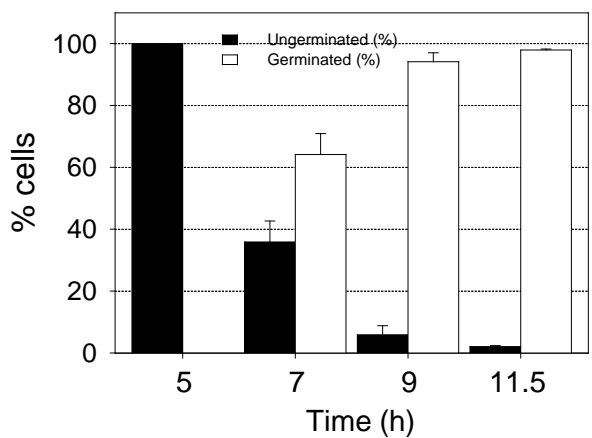

AEQ<sup>CEA10</sup>

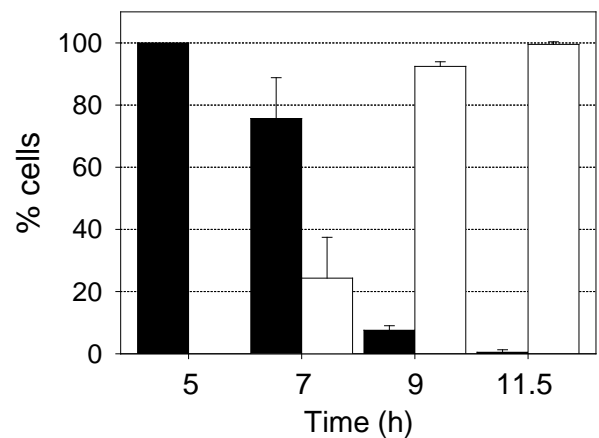

$\DeltaakuB^{KU80}$

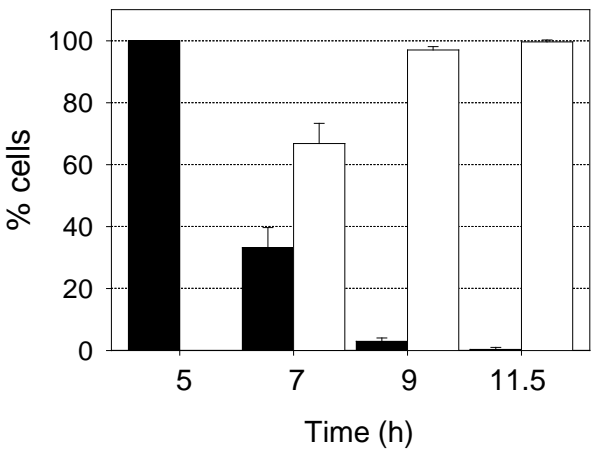

AEQ <sup>$\DeltaakuB^{KU80}$</sup>

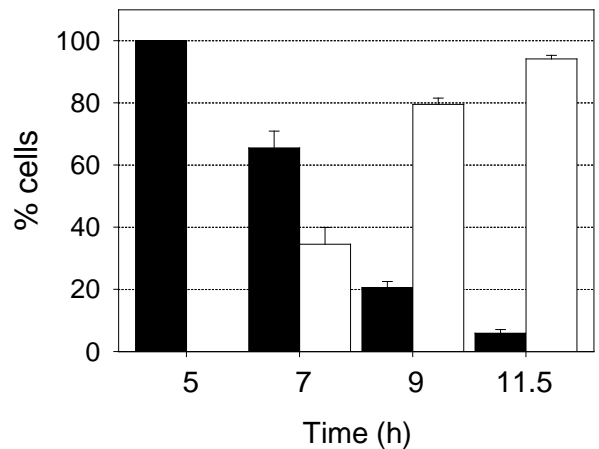

Supplement: S4 Fig — Percentages of ungerminated and germinated conidia were microscopically quantified at different times (5 h to 11.5 h) whilst incubated in AMM at 37°C. (PDF) [file pone.0138008.s005.pdf]

S5 Fig.

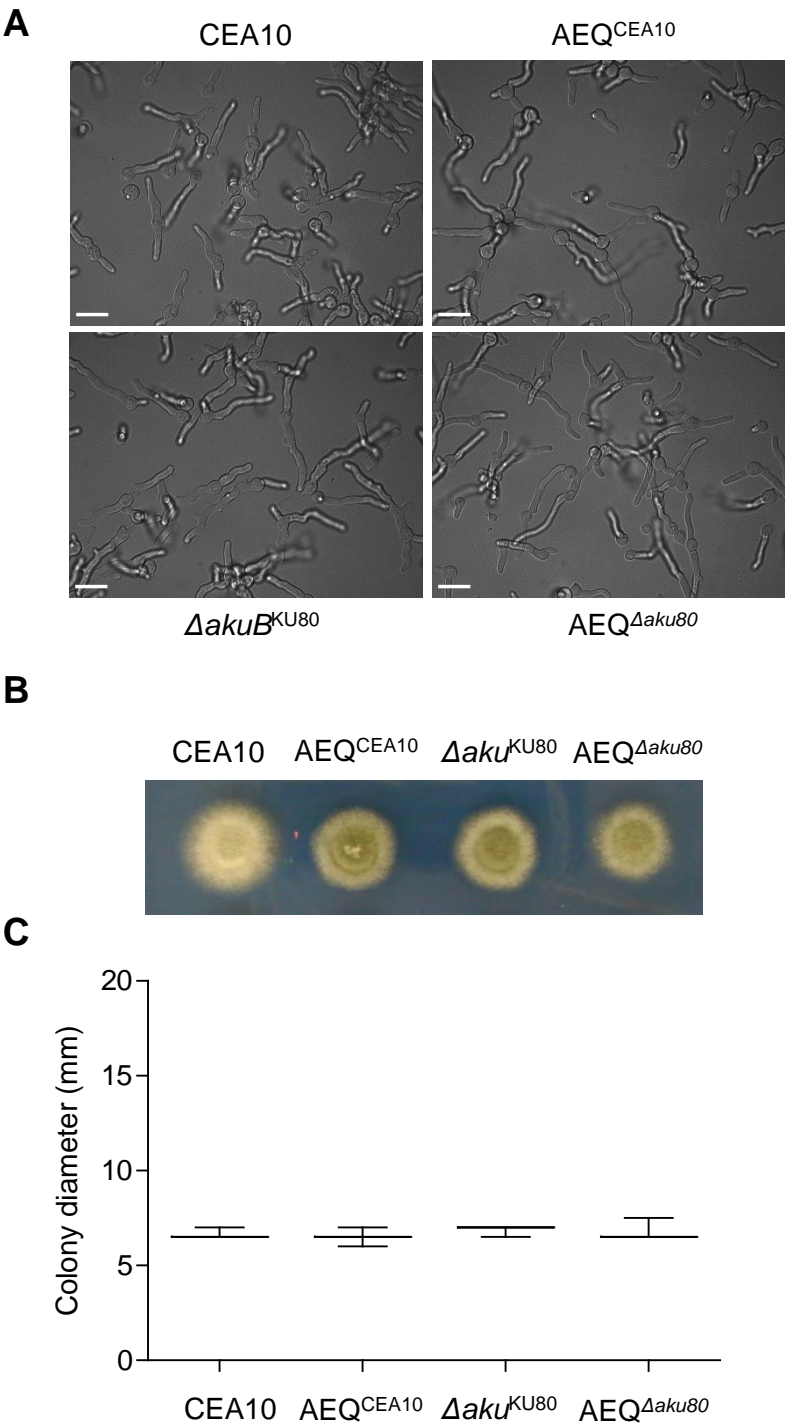

Supplement: S5 Fig — (A) Microscopic visualization of germinated conidia and germlings of the parental and transformant strains after 11.5 h of incubation at 37°C. (B) Radial growth phenotype on solid agar plates after 48 h of incubation at 37°C and (C) colony diameter measurements (in mm). Bar: 10 μm. (PDF) [file pone.0138008.s006.pdf]

S6 Fig.

**A**

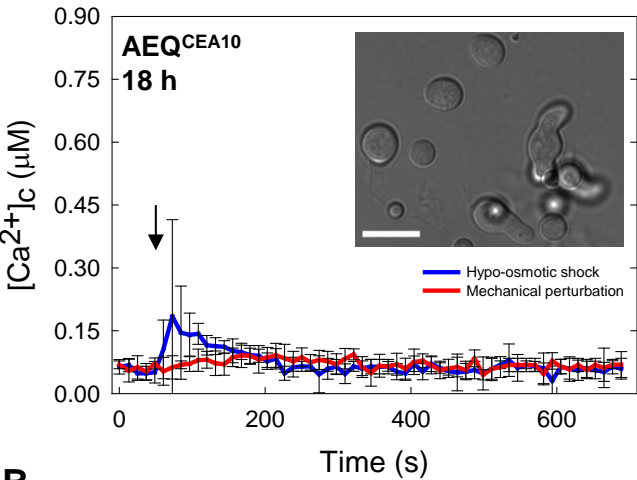

**B**

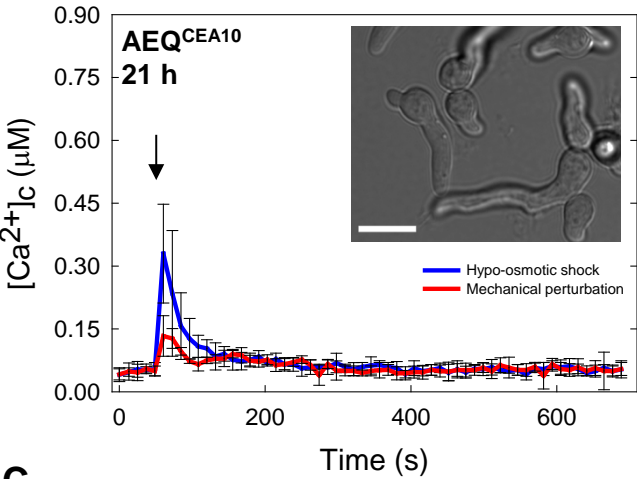

**C**

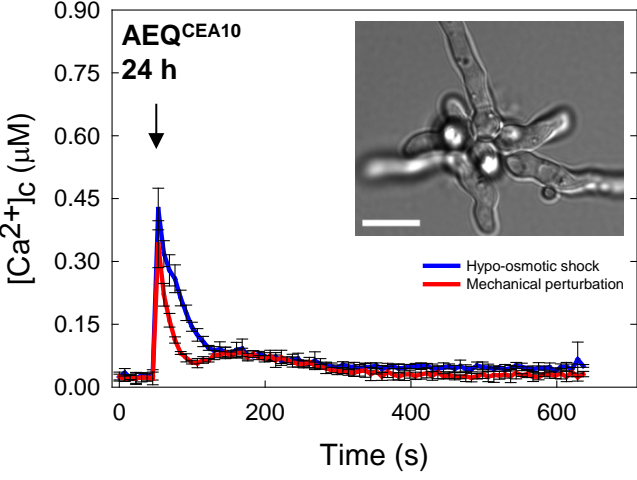

Supplement: S6 Fig — (PDF) [file pone.0138008.s007.pdf]

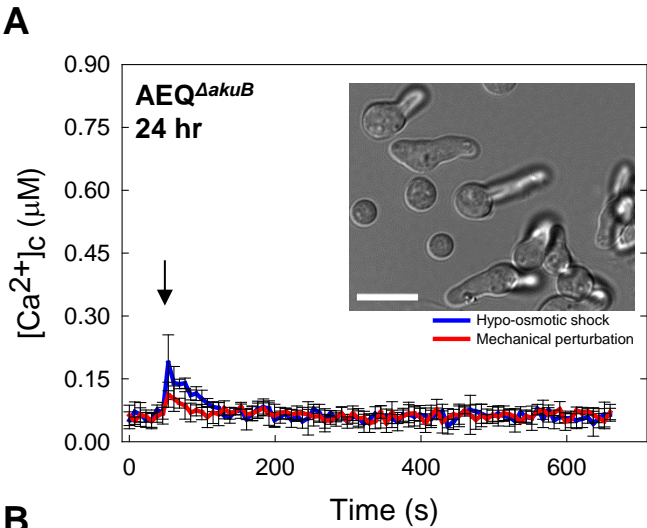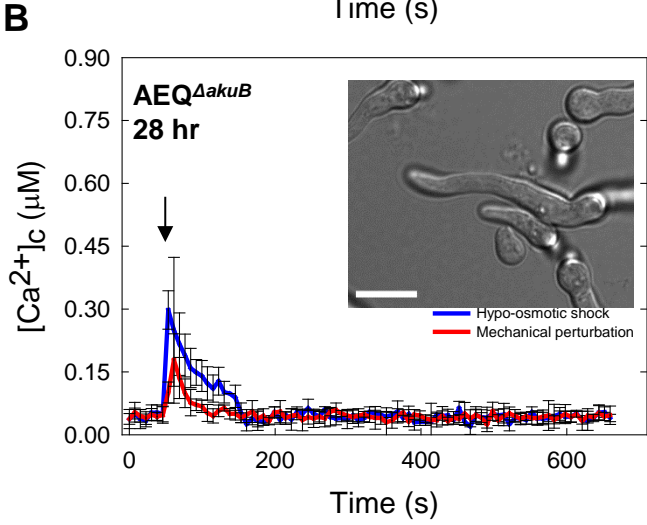

Supplement: S7 Fig — Each stressor was applied at two time-points of growth (24 and 28 h) at 25°C. Cultures were also microscopically analysed in order to compare the stage of conidial germination and germ tube growth with the [Ca2+]c response. Average values ± SD error for six technical replicates are shown. The arrows indicate the point at which each stress was applied via the injectors of the plate reader. Bar: 10 μm. (PDF) [file pone.0138008.s008.pdf]

S8 Fig.

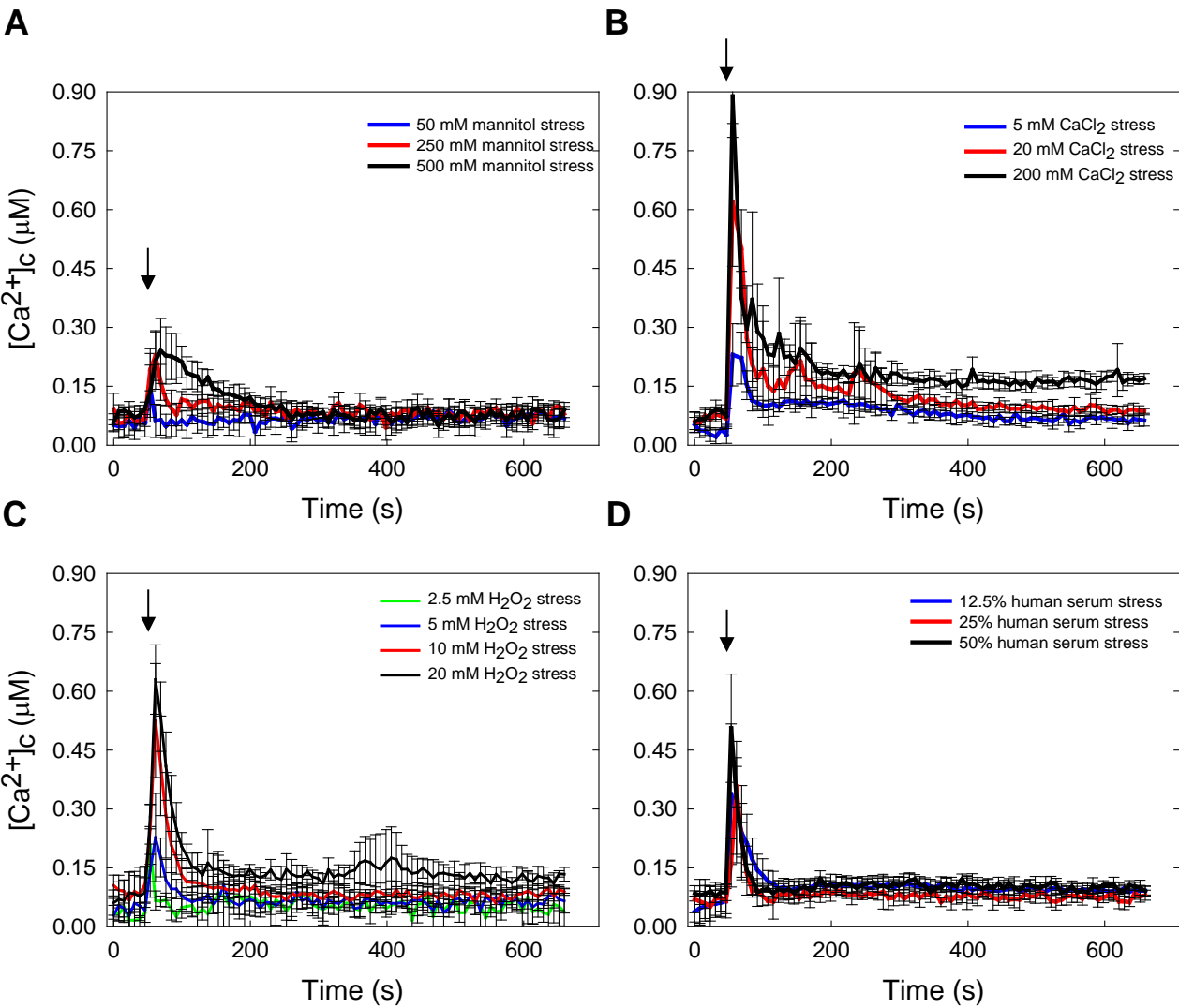

Supplement: S8 Fig — (PDF) [file pone.0138008.s009.pdf]

S9 Fig.

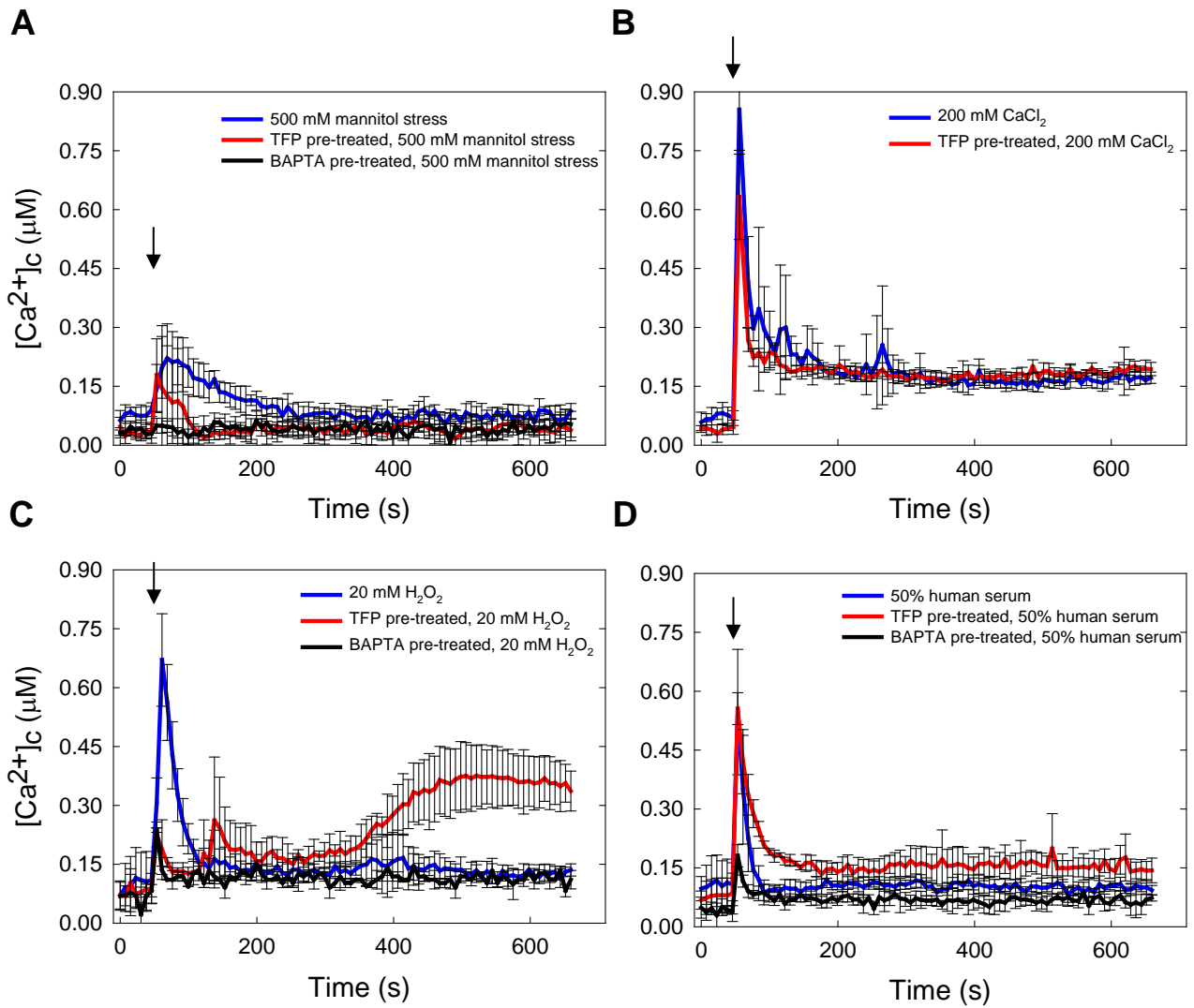

Supplement: S9 Fig — A. fumigatus AEQCEA10 cultures were pre-treated for 30 min with either 5 mM BAPTA or 50 μM TFP prior to challenge with stressors (applied at points indicated by arrows). Note the influence of the modulators on the [Ca2+]c amplitudes and post-stimulatory [Ca2+]c resting levels. Statistical comparisons between untreated and treated samples are presented in Fig 4. (PDF) [file pone.0138008.s010.pdf]
