# Supplementary material for: Different Stress-Induced Calcium Signatures Are Reported by Aequorin-Mediated Calcium Measurements in Living Cells of Aspergillus fumigatus
Source: PLoS One. 2015 Sep 24;10(9):e0138008. doi: 10.1371/journal.pone.0138008 (PMC4581630; doi:10.1371/journal.pone.0138008)
Supplement: S1 Table — (PDF) [file pone.0138008.s011.pdf]

TABLE S1. Effect of the available aequorin on the $[Ca^{2+}]_c$ resting level

Strain	Conditions	Total RLUs	Average resting $[Ca^{2+}]_c$ (µM)	SD
AEQ$^{\Delta akuB}$ 2.8	AMM (5 mM uracil, 10 mM uridine) – 28˚C – 18 h	624093	0.464	0.053
AEQ$^{\Delta akuB}$ 2.4	AMM (5 mM uracil, 10 mM uridine) – 28˚C – 18 h	938017	0.326	0.085
AEQ^{CEA10} 5	AMM – 25˚C – 18 hr	961793	0.449	0.079
AEQ$^{\Delta akuB}$ 2.6	AMM (5 mM uracil, 10 mM uridine) – 28˚C – 18 h	1055935	0.332	0.061
AEQ$^{\Delta akuB}$ 2.10	AMM (5 mM uracil, 10 mM uridine) – 28˚C – 18 h	1065205	0.324	0.088
AEQ$^{\Delta akuB}$ 2.9	AMM (5 mM uracil, 10 mM uridine) – 28˚C - 18 h	1160440	0.304	0.058
AEQ$^{\Delta akuB}$ 2.11	AMM (5 mM uracil, 10 mM uridine) – 28˚C – 18 h	1180644	0.303	0.074
AEQ$^{\Delta akuB}$ 2.9	AMM (5 mM uracil, 10 mM uridine) – 28˚C – 18 h	1731557	0.252	0.049
AEQ$^{\Delta akuB}$ 2.1	AMM (5 mM uracil, 10 mM uridine) – 25˚C – 21 h	1939192	0.226	0.054
AEQ$^{\Delta akuB}$ 2.1	AMM (5 mM uracil, 10 mM uridine) – 28˚C – 18 h	2038444	0.237	0.044
AEQ$^{\Delta akuB}$ 2.1	AMM (5 mM uracil, 10 mM uridine) – 28˚C – 18 h	2637253	0.211	0.049
AEQ^{CEA10} 5	AMM – 28˚C – 18 h	3097941	0.228	0.043
AEQ^{CEA10} 5	AMM – 28˚C – 18 h	3146863	0.260	0.045

$AEQ^{\Delta akuB}$	2.1	AMM (25 mM uracil, 50 mM uridine) – 25˚C - 24h	3193179	0.138	0.055
$AEQ^{\Delta akuB}$	2.1	AMM (5 mM uracil, 10 mM uridine) – 28˚C – 18 h	3455570	0.178	0.033
$AEQ^{\Delta akuB}$	2.1	AMM (25 mM uracil, 50 mM uridine) – 25˚C – 24 h	3543011	0.135	0.038
AEQ^{CEA10}	5	AMM – 28˚C – 18 h	3616656	0.194	0.029
AEQ^{CEA10}	5	AMM – 28˚C – 18 h	3854462	0.176	0.037
$AEQ^{\Delta akuB}$	2.9	AMM (5 mM uracil, 10 mM uridine) – 28˚C – 18 h	4015167	0.161	0.047
AEQ^{CEA10}	5	AMM – 25˚C – 21 h	12224360	0.089	0.028
$AEQ^{\Delta akuB}$	2.1	AMM (5 mM uracil, 10 mM uridine) – 25˚C – 24 h	13909430	0.056	0.025
AEQ^{CEA10}	5	AMM – 25˚C – 21 h	15003427	0.074	0.029
AEQ^{CEA10}	5	AMM – 25˚C – 21 h	15831152	0.083	0.017
AEQ^{CEA10}	5	AMM – 25˚C – 24 h	15924636	0.058	0.020
AEQ^{CEA10}	5	AMM – 28˚C – 18 h	16456851	0.064	0.021
AEQ^{CEA10}	5	AMM – 28˚C – 18 h	19937514	0.068	0.017
AEQ^{CEA10}	5	AMM – 25˚C – 24 h	20607932	0.069	0.028
AEQ^{CEA10}	5	AMM – 28˚C – 18 h	29573819	0.039	0.022
AEQ^{CEA10}	5	AMM – 28˚C – 18 h	34441942	0.032	0.013

AEQ^{CEA10} 5	AMM – 25˚C – 21 h	36856896	0.033	0.023
AEQ^{CEA10} 5	AMM - 28˚C – 20 h	37253064	0.021	0.015
AEQ^{CEA10} 5	AMM - 25˚C – 24 h	39650605	0.032	0.028
AEQ^{CEA10} 5	AMM - 28˚C – 21 h	42805129	0.040	0.036
AEQ^{CEA10} 5	AMM - 28˚C – 18 h	55274954	0.045	0.035
AEQ^{CEA10} 5	AMM - 25˚C – 21 h	73901209	0.021	0.026

AMM: *Aspergillus* minimal medium

SD: standard deviation
